# Exercise Therapy for Chronic Ankle Instability: Which Modality for Which Deficit? A Systematic Review and Meta‐Analysis

**DOI:** 10.1002/jfa2.70142

**Published:** 2026-03-02

**Authors:** Jia Sheng Xu, Hui Juan Lin, Zhi Kun Li, Zi Long Wang, Chao Fan, Hui Fang Chen, Di Xie

**Affiliations:** ^1^ University of Sports and Health Guangzhou Sport University Guangzhou China; ^2^ Guangdong Provincial Key Laboratory of Intelligent Sports and Mental Health Guangzhou China; ^3^ Guangdong Key Laboratory of Human Sports Performance Science Guangzhou China; ^4^ University of Xiamen Xiamen China; ^5^ University of Guangzhou Medical Guangzhou China

**Keywords:** ankle instability, clinical framework, exercise intervention

## Abstract

**Objective:**

Chronic ankle instability (CAI) involves heterogeneous sensorimotor deficits. Evidence comparing exercise modalities for specific deficits is limited, impeding personalised rehabilitation. To determine the relative efficacy of exercise interventions and create an evidence‐based framework for deficit‐specific prescription in CAI.

**Design:**

Systematic review and meta‐analysis.

**Data Sources:**

Searches of 9 electronic databases from inception to 1 September 2025.

**Study Selection:**

Randomised controlled trials comparing exercise interventions against no intervention for sensorimotor outcomes in CAI individuals.

**Data Extraction and Synthesis:**

Two reviewers independently conducted the screening, and will independently extract the data into an Excel spreadsheet. Methodological quality was assessed using PEDro criteria, and bias analysis was conducted using the Cochrane Risk of Bias tool 2. Sensitivity analysis and *I* ^2^ statistics evaluated heterogeneity. Meta‐analysis summarised standard mean difference for sensorimotor indicators.

**Results:**

Fifty‐eight studies (*n* = 2097) were included. Of the included studies, 28 were rated as low risk of bias, 19 as unclear risk and 11 as high risk. Balance training provided comprehensive benefits, improving patient‐reported function, dynamic balance, joint position sense, concentric strength and functional performance. Strength training enhanced patient‐reported function, dynamic balance and concentric strength. 3D, stroboscopic and neuromuscular training improved patient‐reported function and dynamic balance; stroboscopic training also benefited joint position sense. Vibration training improved only dynamic balance. No interventions significantly improved force sense or eccentric strength.

**Conclusion:**

Exercise modalities have distinct efficacy profiles in CAI. Balance training is foundational, but therapy can be personalised by framework optimises management.

**Trial Registration:**

This systematic review was registered in the PROSPERO database (CRD42024606683)

## 1 Introduction

Lateral ankle sprains (LAS) rank among the most prevalent musculoskeletal injuries in physically active populations and exhibit substantial frequency within the general population [1]. Notably, it is estimated that approximately 40% of individuals experiencing LAS develop chronic ankle instability (CAI) [2]. Critically, CAI represents a multifactorial condition, with dysfunction within the sensorimotor system constituting a fundamental pathomechanical underpinning. Compelling evidence indicates that individuals with CAI exhibit consistent deficits across key sensorimotor domains, including postural control (balance), joint position sense, muscular strength and reaction time [3]. Consequently, effectively addressing sensorimotor deficits emerges as a paramount therapeutic objective in the management of CAI.

Given the centrality of sensorimotor system impairment in CAI pathology, the value of exercise therapy is particularly pronounced in remediating these core deficits [4, 5, 6]. Evidence supports the efficacy of targeted exercise paradigms, which could enhance patient‐reported functional impairment and stability and mitigate the progression or symptoms (pain and swelling) of CAI [4], with proprioceptive training demonstrating comparable efficacy to strength training for functional improvement [7], specific enhancements in dynamic postural control [8] and neuromuscular training (e.g., PNF) yield significant gains in multidirectional balance, muscle strength and pain reduction [9]. The meta‐analysis by Guo et al. [10] focused solely on the impact of balance training on patient‐reported functional impairment and stability and dynamic balance (*n* = 341), is constrained by its examination of a singular intervention and limited outcome parameters. Beyond traditional modalities, emerging exercise forms demonstrate promising potential: for instance, research suggests vibration training could ameliorate various sensorimotor indices in patients with CAI, encompassing balance, strength, joint position sense and muscle activation patterns [11]. Similarly, innovative three‐dimensional (3D) training programs (e.g., Tai Chi and dance) aimed at enhancing neuromuscular control through multiplanar movement exposure are gaining research attention. Yin et al. [12] confirmed that Tai Chi could improve dynamic balance, muscular strength and joint position sense in individuals with CAI. Moreover, stroboscopic vision training, which enhances dynamic balance and proprioception by reducing visual input for participants, has also emerged as one of the novel forms of exercise interventions. Although preliminary studies report its benefits on landing mechanics and postural control [13], there is currently no systematic evidence summarising its efficacy for individuals with CAI.

In conclusion, the majority of existing studies confine their scope to evaluating the effects of specific or limited exercise modalities, failing to provide a comprehensive assessment of the multidimensional effects across the entire sensorimotor system. Furthermore, it is unclear which type of intervention (such as physical therapy or exercise) is the most effective for certain sensory or motor impairments. This gap hinders the formulation of evidence‐based recommendations for optimal intervention selection tailored to specific deficit profiles. Therefore, robust evidence guiding clinicians in selecting the most appropriate exercise intervention based on an individual patient's specific functional impairment profile remains substantially lacking.

Therefore, to address this critical gap, this systematic review and meta‐analysis aims to (1) quantitatively synthesise the comparative efficacy of major exercise interventions (e.g., balance, strength, vibration and 3D training) across the entire spectrum of sensorimotor domains in CAI and (2) based on the findings, propose an evidence‐based clinical framework to guide the selection of the most appropriate exercise intervention tailored to the patient's specific deficit profile.

## 2 Methods

This systematic review was reported according to the Preferred Reporting Items for Systematic Reviews and Meta‐Analyses (PRISMA) 2020 statement [14]. Where possible, the methodological recommendations by the Cochrane Collaboration were followed. This review was prospectively registered in PROSPERO with the ID CRD42024606683, and the conduct of the review strictly adhered to the protocol. There was no patient or public involvement.

### 2.1 Data Sources and Searches

A comprehensive systematic search from the inception of the databases to September 1, 2025, across PubMed, Proquest, Web of Science, Cochrane, Embase, Scopus, CNKI, Wanfang and VIP. In addition to the database searches, citation tracking was employed to identify additional eligible studies. The comprehensive search strategy is detailed in Supporting Information S1: Method S1. When data were unavailable or missing, attempts were made to contact the corresponding authors to obtain the data.

### 2.2 Eligibility Criteria

The eligibility criteria were established based on the PICOS framework, which stands for Participants, Interventions, Comparator, Outcomes, and Study design.

Participants (P) individuals who were explicitly defined as having ankle instability/chronic ankle instability/functional ankle instability were included in the study. To ensure the accuracy of the experimental results, individuals with mechanical ankle instability due to anatomical damage or healthy individuals were excluded.

Interventions (I) commonly employed exercise interventions in the domain of ankle instability were included, encompassing balance training, strength training, vibration training and others (corrective training and sensorimotor training). Notably, dance and Tai Chi were categorised as 3D training modalities in accordance with the classification proposed by Kendrick et al. [15]. Multicomponent interventions or nonexercise intervention types (such as surgery and pharmacological treatment) and interventions less than 4 weeks in duration were excluded.

Comparator (C) single exercise interventions were compared with no intervention/placebo or an uninjured limb, and comparisons among three groups were permitted (e.g., balance training vs. strength training vs. no intervention). Studies were excluded if no eligible control group was available.

Outcome (O) studies were included if participants show no significant differences at baseline and the study reports at least one indicator of interest, including patient‐reported functional impairment and stability (Cumberland Ankle Instability Tool (CAIT) and Ankle Joint Functional Assessment Tool (AJFAT)), joint position sense (inversion), pain, dynamic balance, peroneus longus muscle reaction time, muscular strength and functional performance. Studies that do not report relevant indicators and those that only conduct pre–post comparisons were excluded.

Study Design (S) to minimise bias in this study, only RCTs were included as RCTs represent the experimental design with the least potential for selection bias [16].

### 2.3 Study Selection

The retrieved literature was reviewed and deduplicated using NoteExpress software (Version V4.X). Initially, two reviewers (JX and HL) independently screened the studies based on their titles and abstracts. Following the preliminary screening, the full texts of all potentially eligible trials were retrieved and reviewed. Any disagreements were resolved through consensus discussions, with a third reviewer (DX) consulted if necessary. All downloaded literature was then imported into Zotero software (Version 7.0.13; Corporation for Digital Scholarship, Vienna, VA) for management.

### 2.4 Data Extraction and Outcome Measures

Two reviewers (JX and ZW) independently extracted relevant information from each included trial, with cross‐validation conducted upon completion. The main data items included: study characteristics (authors, year, type of intervention and measurement methods), participant characteristics (age, gender and number of participants per group) and primary outcomes (outcome measures and statistical significance). The extracted data were compiled into tables. Any disagreements that could not be resolved were adjudicated by a third reviewer (DX).

For trials involving two types of interventions, data were extracted and categorised under their respective intervention types. For studies with repeated measurements at multiple time points, data were categorised and compared across these time points (e.g., 4, 6 and 8 weeks). For the primary analysis, the time point closest to, but not exceeding, the 4–6 week postintervention window was selected. If multiple assessments were available within this same designated window, the earliest measurement was chosen for analysis. Different measurement methods (e.g., dynamic balance tests such as Star Excursion Balance Test) (SEBT) and modified Star Excursion Balance Test (mSEBT) and units of measurement (e.g., functional performance tests measured in metres and seconds) for the same indicator were also categorised. In accordance with Cochrane guidelines [17], outcomes measured using multiple parameters within the same study were pooled using standardised methods.

If the study mentioned the indicators of interest but did not report them or had partial missing data in the results, the authors were contacted via email up to three times to obtain the information. If the data could not be obtained after these attempts, the study was excluded.

### 2.5 Assessment of Risk of Bias

To assess the reliability and validity of the included studies, two authors independently conducted assessments of bias risk. The Cochrane Risk of Bias 2.0 (RoB 2.0) tool [18] was used to evaluate RCTs, which assesses the studies in five areas: D1 (randomisation and generation of sequences), D2 (allocation concealment), D3 (outcome assessment), D4 (completeness of result data) and D5 (selective reporting of results). Funnel plots were generated and Egger's test was performed to assess small‐study effects when more than 10 studies were included for a given outcome. To assess the robustness of the results, sensitivity analyses were conducted for all indicators using Stata 18.0 software.

### 2.6 Assessment of Study Quality

The methodological quality assessment was conducted using the Physiotherapy Evidence Database (PEDro) scoring criteria [19], with scores interpreted as follows: less than 4 points indicating poor quality, 4–5 points indicating fair quality, 6‐8 points indicating good quality and 9–10 points indicating excellent quality. Two authors (JX and HL) independently assessed the quality of the articles. Discrepancies in assigned scores were resolved by a senior expert who reviewed the article independently.

### 2.7 Certainty of Evidence

The certainty of evidence was assessed and reported using the GRADE framework. This assessment was performed both for the overall body of evidence and within prespecified subgroups, categorised according to the type of intervention.

### 2.8 Data Synthesis and Statistical Analysis

All indicators were continuous variables, and the mean and standard deviation (SD) were used for meta‐analysis. The main outcomes were divided into sensory and motor function indicators. The *I* ^2^ statistic was used to test for statistical heterogeneity, with low heterogeneity defined as *I* ^2^ < 40%, moderate heterogeneity as 30%–60%, significant heterogeneity as 50%–90% and high heterogeneity as 75%–100% [18]. The *I* ^2^ and Q statistics (*p* < 0.1) were calculated to assess the heterogeneity of the studies included in the analysis. Based on the degree of heterogeneity, the fixed‐effect model or random‐effects model was applied to analyse the included studies. In the random‐effects meta‐analyses, between‐study variance (*τ* ^2^) was estimated using the DerSimonian–Laird method and Wald‐type confidence intervals were calculated for the pooled effect estimates.

Cohen's *d* effect sizes along with their standard errors were calculated using the pooled standard deviation across groups for the main outcomes, to ascertain the magnitude of differences between groups. Effect size estimates were interpreted as follows: negligible (less than 0.20), small (0.21–0.39), medium (0.40–0.79) and large (more than 0.80) effect size categories [20]. If at least three studies reported the same outcome, a meta‐analysis was conducted and the impact of inconsistent units was addressed using a random‐effects model. The statistical analysis was performed using Review Manager (Version 5.4, 2020) to evaluate outcomes between the intervention and control groups. In accordance with Cochrane guidelines, appropriate adjustments were made to the sample sizes and standard deviations for studies involving a shared control group. The Stata 18.0 software was employed to generate the sensitivity analysis plots for each outcome indicator, the impact on the effect size (95% confidence intervals) of the meta‐analysis for each indicator was assessed by the method of leave‐one‐out sensitivity analysis.

### 2.9 Development of the Clinical Framework

Based on the results of the meta‐analysis, a clinical decision framework was developed iteratively by the authorship team. The recommendations within the framework are directly derived from the statistically significant findings (*p* < 0.05) and the magnitude of the effect sizes (SMD) for each intervention‐outcome pairing. Interventions with large effect sizes and consistent significant outcomes were prioritised as primary recommendations.

## 3 Results

### 3.1 Literature Search and Screening

**FIGURE 1:**
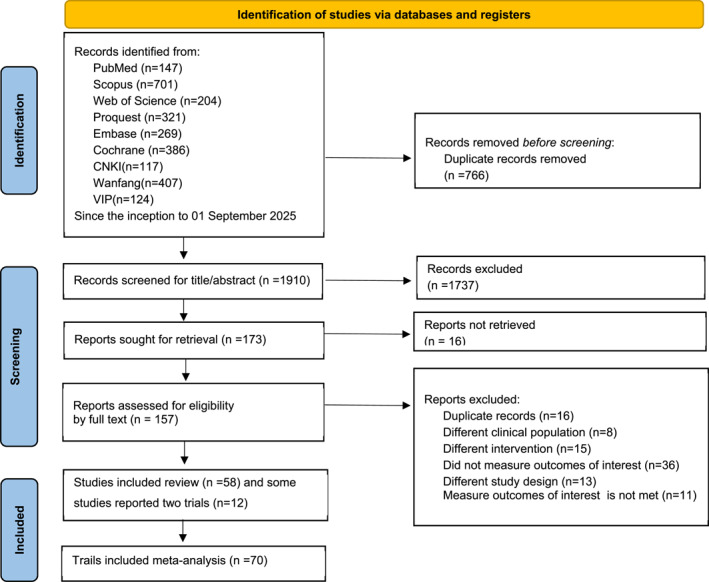
PRISMA flowchart for study selection.

The systematic search identified 2676 records. Following removal of 766 duplicates, 1910 unique records underwent title/abstract screening. Of these, 173 studies were deemed eligible for full‐text assessment. Ultimately, 58 studies 21, 22, 23, 24, 25, 26, 27, 28, 29, 30, 31, 32, 33, 34, 35, 36, 37, 38, 39, 40, 41, 42, 43, 44, 45, 46, 47, 48, 49, 50, 51, 52, 53, 54, 55, 56, 57, 58, 59, 60, [61, 62, 63, 64, 65, 66, 67, 68, 69, 70, 71, 72, 73, 74, 75, 76, 77, 78] met the inclusion criteria; however, 12 multiarm studies contributed data to two distinct intervention categories, resulting in 70 independent trial datasets for quantitative synthesis (Figure 1).

### 3.2 Characteristics of the Eligible Studies

The study included a total of 2097 participants with ankle instability; the characteristics of all included studies are detailed in Supporting Information S1: Table S1.

A total of 21 studies reported on balance training, nine studies on vibration training, 14 studies on strength training, seven studies on neuromuscular control training, four studies on proprioceptive training, three studies on stroboscopic vision training and nine studies on 3D training (Tai Chi and dance). Additionally, two studies reported on corrective training and sensorimotor training (other). Among them, 54 trials reported outcome measures for sensory outcomes and 25 trials reported outcome measures for the motor system.

### 3.3 Outcomes of Risk of Bias Assessment

**FIGURE 2:**
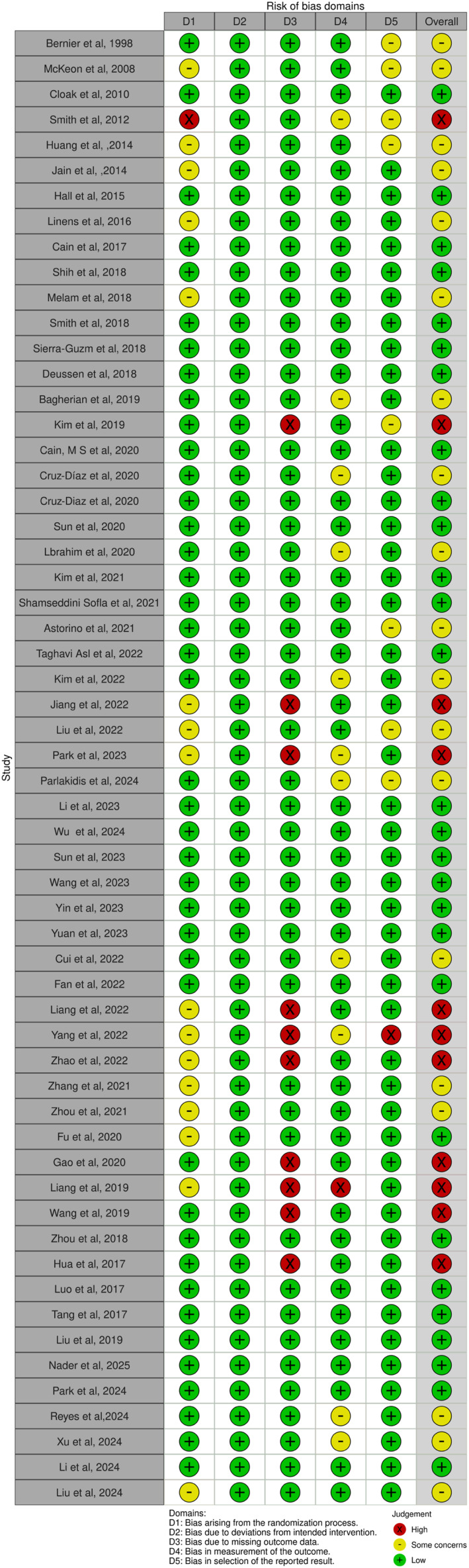
Risk of bias plot.

The bias analysis results showed that 28 articles were at low risk of bias, 19 articles were at medium risk of bias and 11 articles were at high risk of bias. Most types of exercise intervention were associated with a low to moderate risk of bias, with the exception of proprioceptive training. The primary reasons for studies being categorised as having a moderate risk of bias were domains D1, D4 and D5, whereas the principal cause for studies being classified as having a high risk of bias was domain D3 (Figure 2).

For outcome measures, including joint position sense, dynamic balance, patient‐reported functional impairment and stability, concentric inversion muscular strength and concentric eversion muscular strength, funnel plots were constructed and Egger's test (Supporting Information S1: Figure S1) was performed. Egger's test results indicated that the *p*‐values for all the aforementioned outcomes were below 0.05 (Supporting Information S1: Table S2).

### 3.4 Outcomes of Quality Assessment

The results of the quality assessment revealed that 12 articles were rated as fair, 37 articles as good and 9 articles as excellent, most exercise intervention types were rated as good, with the exception of those investigating proprioceptive training. Studies rated as being of fair quality were primarily due to insufficient rigor in the study design, particularly regarding blinding (Supporting Information S1: Table S3).

### 3.5 Outcomes of Certainty of Evidence

According to the GRADE assessment, the certainty of evidence was rated as low for functional performance (based on the time to complete the test), pain and force sense; as moderate for joint position sense, concentric inversion muscular strength and patient‐reported functional impairment and stability and as high for all remaining outcome measures. For detailed ratings, please refer to Supporting Information S1: Table S4.

### 3.6 Assessment of Heterogeneity

The overall heterogeneity assessment results of the exercise intervention on various indicators are as follows: the patient‐reported functional impairment and stability (*χ* ^2^ = 104.15, *p* < 0.1 and *I* ^2^ = 71%), the joint position sense (*χ* ^2^ = 43.17, *p* < 0.1 and *I* ^2^ = 77%), pain (*χ* ^2^ = 3.45, *p* = 0.18 and *I* ^2^ = 42%), force sense (*χ* ^2^ = 3.00, *p* = 0.22 and *I* ^2^ = 33%), dynamic balance (*χ* ^2^ = 95.52, *p* < 0.1 and *I* ^2^ = 45%), muscle reaction time (*χ* ^2^ = 0.38, *p* = 0.83 and *I* ^2^ = 0%), functional performance (completion time) (*χ* ^2^ = 145.37, *p* < 0.1 and *I* ^2^ = 94%), functional performance (jump stance) (*χ* ^2^ = 1.53, *p* = 0.68 and *I* ^2^ = 0%), muscular strength (concentric inversion muscular strength) (*χ* ^2^ = 31.68, *p* = 0.03 and *I* ^2^ = 40%), muscular strength (eccentric inversion muscular strength) (*χ* ^2^ = 5.06, *p* = 0.17 and *I* ^2^ = 41%), muscular strength (concentric eversion muscular strength) (*χ* ^2^ = 71.57, *p* < 0.1 and *I* ^2^ = 71%) and muscular strength (eccentric eversion muscular strength) (*χ* ^2^ = 6.74, *p* = 0.24 and *I* ^2^ = 26%).

### 3.7 Sensitive Analysis

No significant shifts (less than 10%) were observed in the effect sizes or confidence intervals for any outcome, indicating that the study findings are robust and stable. Furthermore, after excluding studies with a high risk of bias based on the risk of bias assessment, no statistically significant change was found in any of the study outcomes. These results indicate that the outcomes of the meta‐analysis are stable and reliable (Supporting Information S1: Figure S2).

### 3.8 Meta‐Analysis Results

**FIGURE 3:**
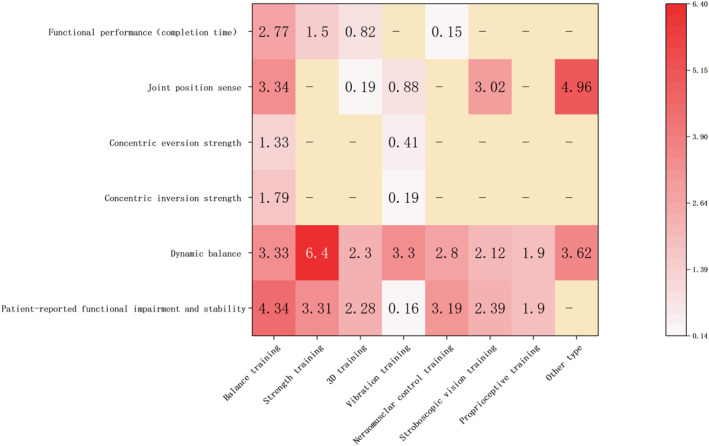
Heat map of efficacy of exercise interventions.

The meta‐analysis revealed significant improvements in patient‐reported functional impairment and stability, joint position sense (inversion), pain, dynamic balance, muscle reaction time, functional performance (completion time), functional performance (jump stance), concentric inversion and eversion muscular strength following exercise interventions. The heatmap is provided in Figure 3. No significant improvements were observed in force sense, eccentric inversion and eversion muscular strength. For the outcome indicators of muscle reaction time, pain and force sense, subgroup analyses were not conducted due to insufficient numbers of studies across different types of exercise interventions.

### 3.9 Pain

**FIGURE 4:**
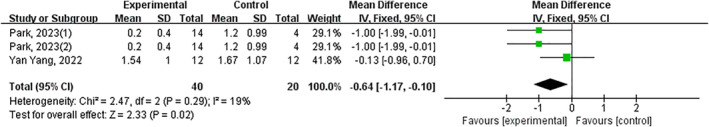
Forest plot‐pain.

Three trials demonstrated significant pain reduction with large effect size (*p* < 0.05, SMD −0.84, 95% CI −1.20–0.4 and ES = 4.61) (Figure 4).

### 3.10 Patient‐Reported Functional Impairment and Stability

**FIGURE 5:**
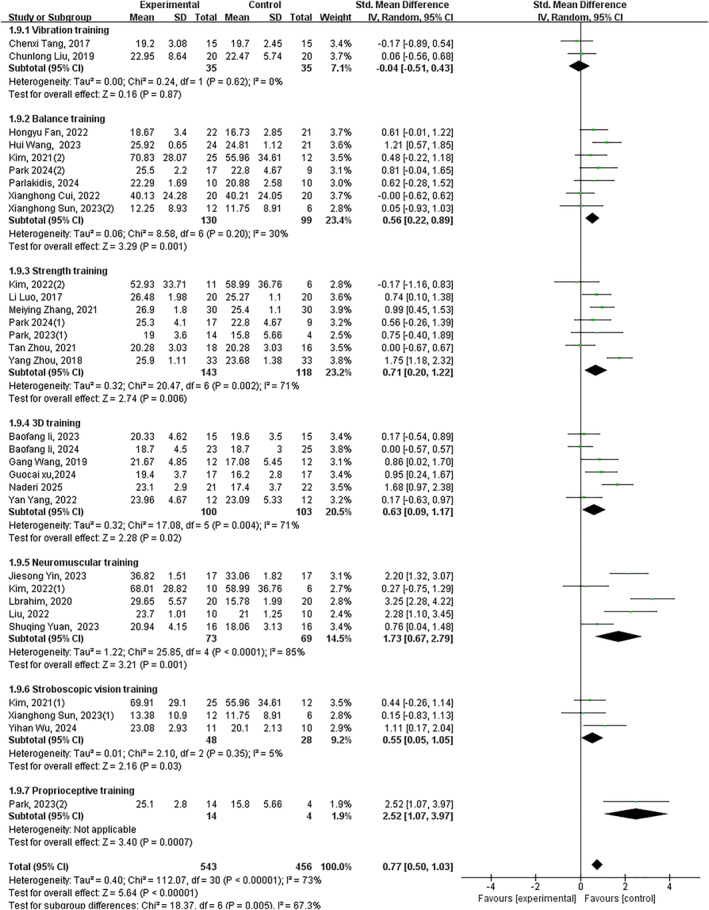
Forest plot‐patient‐reported functional impairment and stability.

Twenty‐six trials demonstrated that exercise intervention significantly improved patient‐reported functional impairment and stability in individuals with CAI, with a large effect size (*p* < 0.05, SMD 0.75, 95% CI 0.51–0.99 and ES = 6.25). Subgroup analysis revealed significant improvements with: balance training (*p* < 0.05, SMD 0.63, 95% CI 0.34–0.9 and ES = 4.34), strength training (*p* < 0.05, SMD 0.76, 95% CI 0.31–1.21 and ES = 3.31), 3D training (*p* < 0.05, SMD 0.63, 95% CI 0.09–1.17 and ES = 2.28), neuromuscular control training (*p* < 0.05, SMD 1.73, 95% CI 0.67–2.79 and ES = 3.19) and stroboscopic vision training (*p* < 0.05, SMD 0.54 95% CI 0.10–0.99 and ES = 2.39). No significant improvements were observed with vibration training (*p* = 0.87) or proprioceptive training (*p* = 0.06) (Figure 5).

### 3.11 Dynamic Balance

**FIGURE 6:**
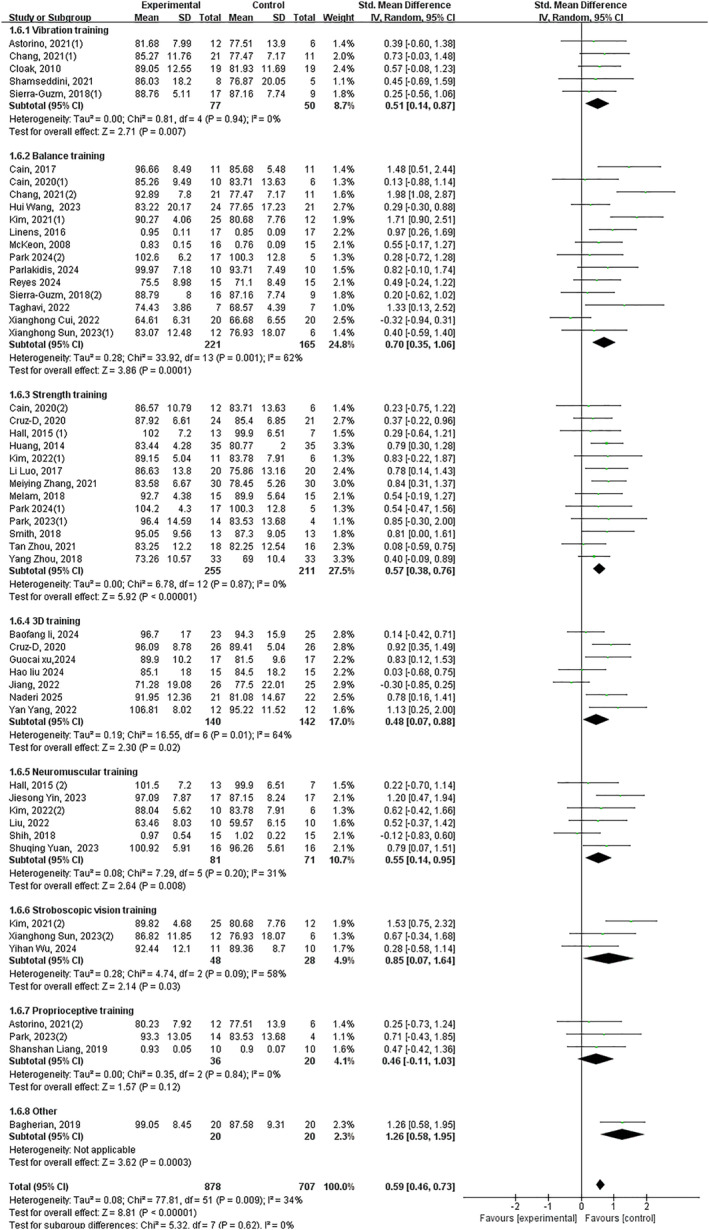
Forest plot‐dynamic balance.

Forty‐two trials revealed significant improvement in dynamic balance (*p* < 0.05, SMD 0.59, 95% CI 0.45–0.72 and ES = 8.57). All interventions showed significant effects except proprioceptive training: vibration training (*p* < 0.05, SMD 0.55, 95% CI 0.22–0.88 and ES = 3.30), balance training (*p* < 0.05, SMD 0.59, 95% CI 0.24–0.94 and ES = 3.33), strength training (*p* < 0.05, SMD 0.59, 95% CI 0.41–0.77 and ES = 6.40), 3D training (*p* < 0.05, SMD 0.48, 95% CI 0.07–0.8 and ES = 2.30), neuromuscular control training (*p* < 0.05, SMD 0.56, 95% CI 0.17–0.95 and ES = 2.80), stroboscopic vision training (*p* < 0.05, SMD 0.97, 95% CI 0.07–1.87 and ES = 2.12) and other interventions (*p* < 0.05, SMD 1.26, 95% CI 0.58–1.95 and ES = 3.62). Proprioceptive training showed nonsignificant improvement (*p* = 0.06) (Figure 6).

### 3.12 Joint Position Sense

**FIGURE 7:**
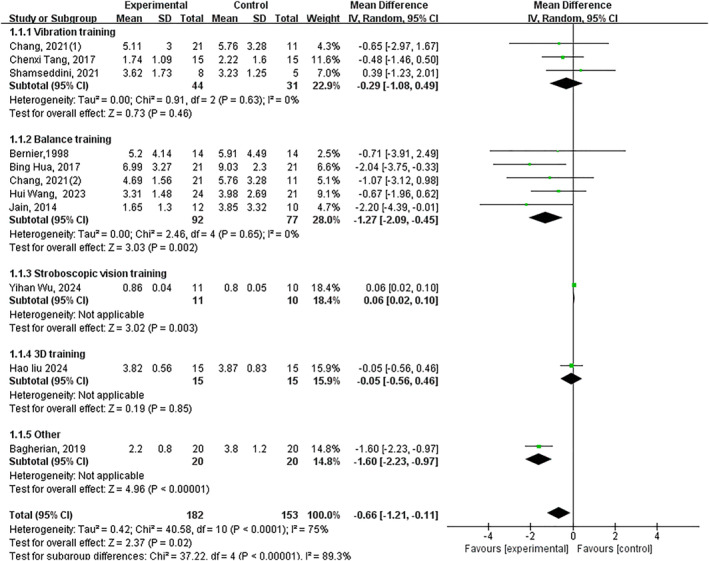
Forest plot‐joint position sense.

Ten trials showed exercise intervention significantly improved joint position sense (*p* < 0.05, SMD −0.68, 95% CI −1.21–−0.15 and ES = 2.51). Significant subgroup effects occurred with: balance training (*p* < 0.05, SMD −1.22, 95% CI −1.94–−0.50 and ES = 3.34), stroboscopic vision training (*p* < 0.05, SMD 0.06, 95% CI 0.02–0.10 and ES = 3.02) and other interventions (*p* < 0.05, SMD −1.60, 95% CI −2.23–−0.97 and ES = 4.96). Nonsignificant effects: vibration training (*p* = 0.38) and 3D training (*p* = 0.85) (Figure 7).

### 3.13 Force Sense

**FIGURE 8:**
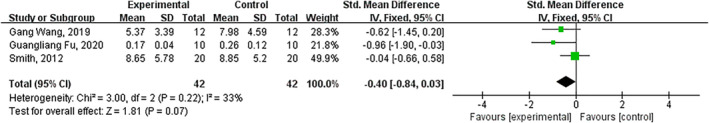
Forest plot‐force sense.

Three trials showed no significant improvement (*p* = 0.07) (Figure 8).

### 3.14 Muscular Reaction Time

**FIGURE 9:**
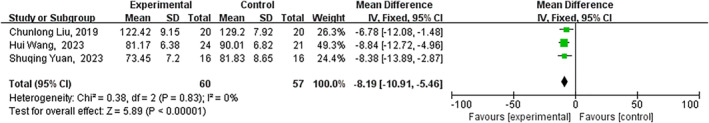
Forest plot‐muscular reaction time.

Three trials demonstrated significantly reduced peroneus longus reaction time (*p* < 0.05, SMD −8.19, 95% CI −10.91–−5.46 and ES = 5.89) (Figure 9).

### 3.15 Inversion Muscular Strength

#### 3.15.1 Concentric (17 Trials)

**FIGURE 10:**
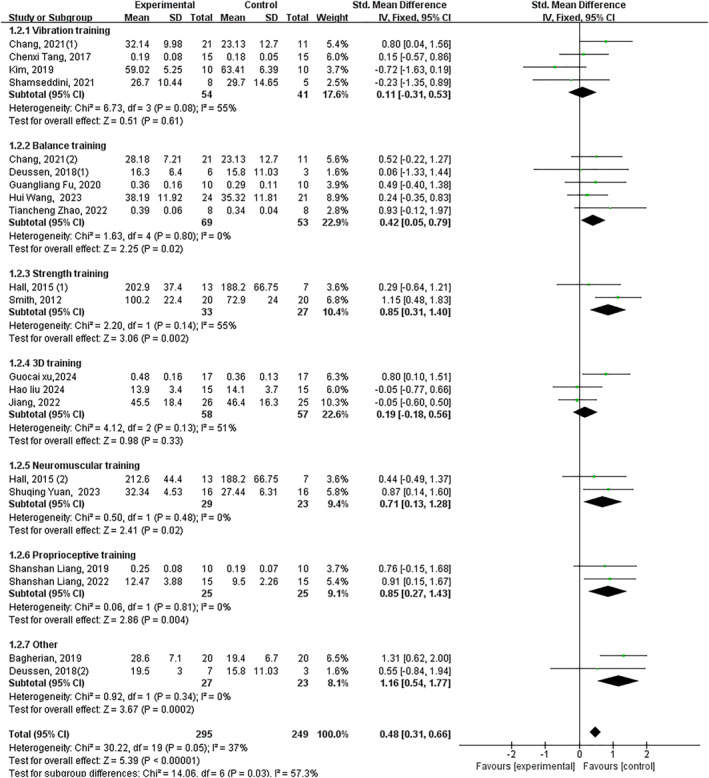
Forest plot‐concentric inversion muscular strength.

Significant improvement overall (*p* < 0.05, SMD 0.51, 95% CI 0.34–0.68 and ES = 5.86). Significant subgroup effects: balance training (*p* < 0.05, SMD 0.45, 95% CI 0.10–0.79 and ES = 2.55), strength training (*p* < 0.05, SMD 0.80, 95% CI 0.29–1.31 and ES = 3.08), neuromuscular training (*p* < 0.05, SMD 0.79, 95% CI 0.18–1.40 and ES = 2.53), proprioceptive training (*p* < 0.05, SMD 0.85, 95% CI 0.27–1.43 and ES = 2.86) and other interventions (*p* < 0.05, SMD 1.11, 95% CI 0.53–1.70 and ES = 3.72). Nonsignificant effects: vibration training (*p* = 0.24) and 3D training (*p* = 0.33) (Figure 10).

#### 3.15.2 Eccentric (3 Trials)

**FIGURE 11:**
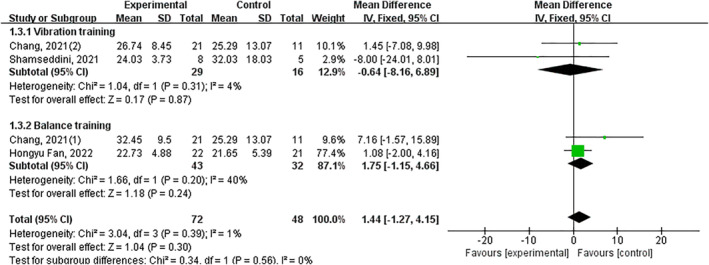
Forest plot‐eccentric inversion muscular strength.

No significant improvement (*p* = 0.10) (Figure 11).

### 3.16 Eversion Muscular Strength

#### 3.16.1 Concentric (18 Trials)

**FIGURE 12:**
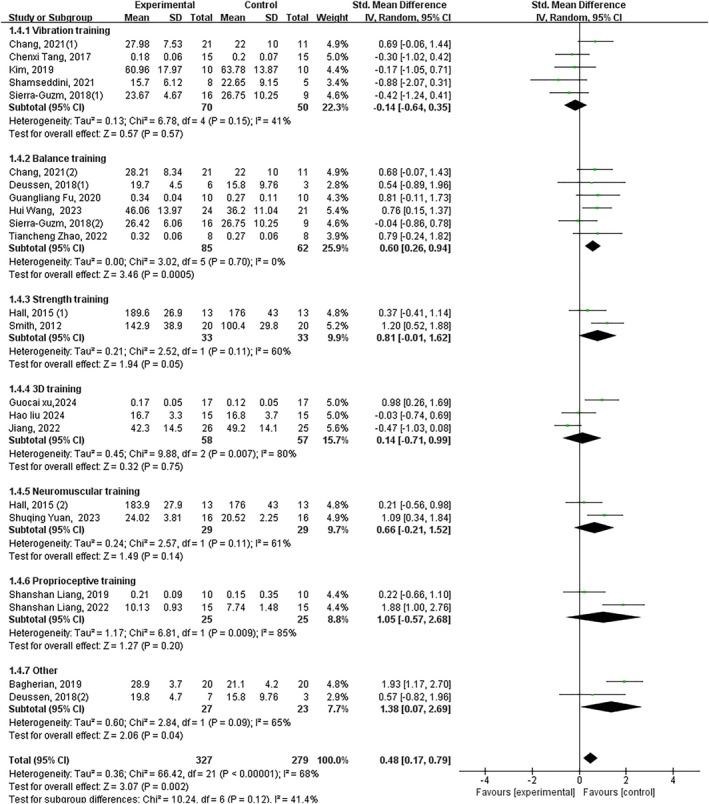
Forest plot‐concentric eversion muscular strength.

Significant improvement overall (*p* < 0.05, SMD 0.49, 95% CI 0.19–0.79 and ES = 3.16). Significant subgroup effects: balance training (*p* < 0.05, SMD 0.60, 95% CI 0.28–0.91 and ES = 3.74), strength training (*p* = 0.05, SMD 0.81, 95% CI −0.01–1.62 and ES = 1.94) and other interventions (*p* < 0.05, SMD 1.16, 95% CI 0.10–2.61 and ES = 2.11). Non‐significant effects: vibration training (*p* = 0.64), 3D training (*p* = 0.75), neuromuscular training (*p* = 0.14) and proprioceptive training (*p* = 0.20) (Figure 12).

#### 3.16.2 Eccentric (3 Trials)

**FIGURE 13:**
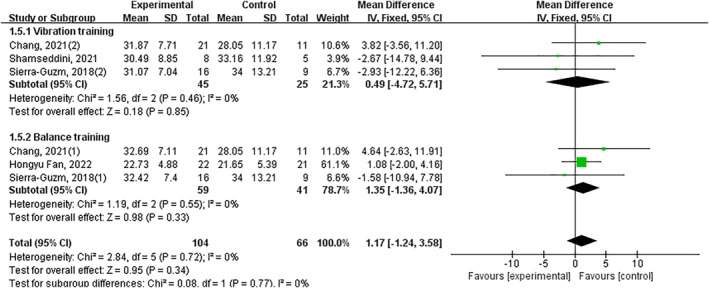
Forest plot‐eccentric eversion muscular strength.

No significant improvement (*p* = 0.18) (Figure 13).

### 3.17 Functional Performance

#### 3.17.1 Completion Time (7 Trials)

**FIGURE 14:**
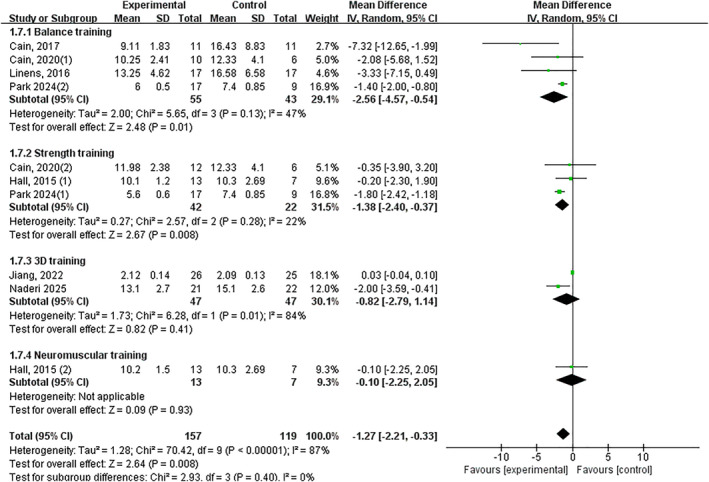
Forest plot‐functional performance (based on the time completing the test).

Significant improvement overall (*p* < 0.05, SMD −1.18, 95% CI −2.03–−0.33 and ES = 2.72), with balance training showing significant effects (*p* < 0.05, SMD •2.33, 95% CI −3.97–−0.68 and ES = 2.77). Nonsignificant effects: strength training (*p* = 0.13), 3D training (*p* = 0.41) and neuromuscular control training (*p* = 0.88) (Figure 14).

#### 3.17.2 Jump Distance (4 Trials)

**FIGURE 15:**
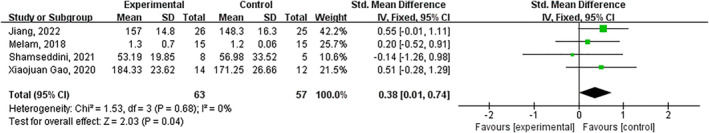
Forest plot‐functional performance (based on the distance completing the test).

Significant improvement (*p* < 0.05, SMD 0.38, 95% CI −0.01–0.74 and ES = 2.03) (Figure 15).

### 3.18 Clinical Framework

**FIGURE 16:**
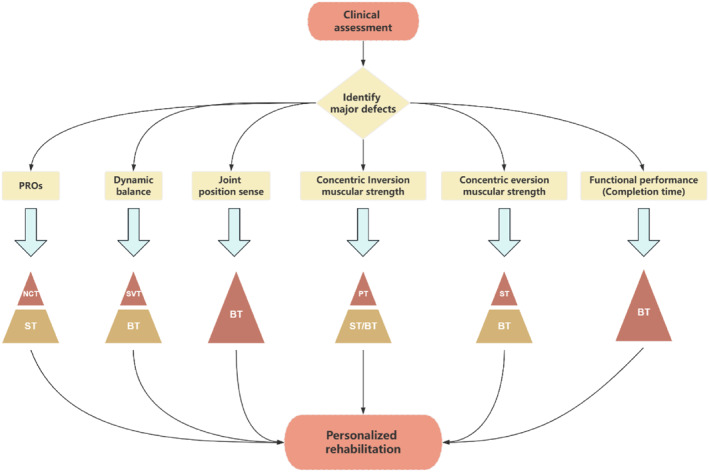
Clinical framework.

Based on the differential efficacy results summarised above, a clinical decision framework for exercise intervention in CAI was constructed (Figure 16).

## 4 Discussion

In this systematic review and meta‐analysis, we found that different forms of exercise intervention exert distinct therapeutic effects on the sensorimotor system in individuals with CAI. Our results indicate that balance training, strength training, 3D training, neuromuscular control training and stroboscopic vision training all significantly improve patient‐reported functional impairment and stability. Among the exercise interventions included in this study, balance training demonstrated the most comprehensive therapeutic effects.

### 4.1 Pain

Pain is a prevalent and clinically significant symptom in CAI, intricately linked to perceived instability and functional limitation [79]. Our meta‐analysis, albeit based on a limited number of trials (*n* = 3), indicates that exercise interventions can collectively induce a significant large‐effect reduction in pain (SMD ‐0.84). This finding is consistent with the broader literature demonstrating that exercise can elevate pressure pain thresholds and modulate pain sensitivity through neurophysiological mechanisms [80]. For instance, Park et al. [49] observed significant pain alleviation following both strength and proprioceptive training. However, the current evidence is insufficient to delineate which specific exercise modality is most effective for pain relief, as subgroup analyses were not feasible. Consequently, although the alleviation of pain should be regarded as a valuable outcome of exercise therapy in general, its utility for guiding the selection of a specific intervention within our clinical decision framework (Figure 16) is presently limited. Future studies with pain as a primary outcome are needed to clarify the differential effects of various exercise types on this important patient‐centred metric.

### 4.2 Patient‐Reported Functional Impairment and Stability

Patient‐reported outcomes (PROs), captured by tools, such as the CAIT and AJFAT, are indispensable for evaluating the subjective experience of instability and functional limitation in CAI, complementing objective performance measures [37]. Our meta‐analysis provides robust evidence that exercise interventions collectively induce a large significant improvement in PROs (SMD 0.75). Critically, subgroup analysis revealed a distinct hierarchy of efficacy. Balance training, strength training, 3D training, neuromuscular control training and stroboscopic vision training all demonstrated significant effectiveness. In contrast, vibration training and proprioceptive training did not produce statistically significant improvements. The broad effectiveness of the former interventions likely stems from their capacity to directly address the underlying pathomechanical and perceptual deficits that contribute to feelings of instability, thereby restoring patient confidence during functional activities. The lack of significant improvement with vibration training may be attributed to intervention parameters (e.g., intensity and frequency) that were insufficient to translate into perceptible functional gains or misaligned with the mechanisms driving subjective instability. The finding for proprioceptive training, based on a single study, requires cautious interpretation and further investigation. Within our proposed clinical decision framework (Figure 16), these results firmly establish balance training as a cornerstone intervention for improving PROs due to its consistent and significant effect. Furthermore, they provide clinicians with a portfolio of effective alternatives (e.g., strength training and 3D training), enabling tailored prescription based on patient preference, resource availability or the need to target concurrent deficits.

### 4.3 Dynamic Balance

Deficits in dynamic postural control, a hallmark of CAI, significantly contribute to the risk of recurrent ankle sprains and diminished functional performance [81, 82]. Our study included the star excursion balance test and calculated the composite value of reach distance in each direction. Consistent with previous studies [11, 83, 84] most forms of exercise intervention could significantly improve the dynamic balance of individuals with CAI. Our analysis, encompassing 42 trials, provides conclusive evidence that exercise interventions collectively induce a moderate‐to‐large, significant improvement in dynamic balance (SMD 0.59). The most compelling finding is the broad efficacy observed across diverse intervention types. Subgroup analyses confirmed that vibration training, balance training, strength training, 3D training, neuromuscular control training and stroboscopic vision training all yielded significant positive effects. This suggests that the fundamental mechanism of improvement may involve a shared pathway of enhancing neuromuscular control and stability under dynamic, challenging conditions that mimic real‐world demands. The singular exception was proprioceptive training, which did not show a statistically significant effect on composite dynamic balance scores, despite isolated reports of improvement in specific directions [44, 49]. This discrepancy may arise from differences in training specificity or the metrics used for evaluation. Within our clinical decision framework (Figure 16), the implications are highly practical. The robust evidence for balance training reinforces its status as a primary intervention for dynamic balance deficits. Crucially, the framework also validates a range of effective alternatives—including vibration training, 3D training and neuromuscular control training—providing clinicians with valuable flexibility to tailor rehabilitation based on equipment access, patient preference or the need to address coexisting impairments.

### 4.4 Proprioception

Proprioceptive deficits, encompassing both joint position sense (JPS) and force sense, are a core impairment in CAI, disrupting the sensorimotor feedback essential for joint stability [85, 86]. Our meta‐analysis reveals a nuanced picture regarding the efficacy of exercise interventions in remediating these deficits. For joint position sense, a large significant overall improvement was observed (SMD ‐0.68). Critically, the effects were driven by specific interventions. Balance training demonstrated a very large effect, likely by repetitively stimulating articular and ligamentous mechanoreceptors under challenging postural conditions, thereby enhancing the central processing of afferent signals [87]. Stroboscopic vision training also showed efficacy, potentially by forcing a sensory reweighting that increases reliance on proprioceptive input when vision is disrupted. In contrast, vibration training and 3D training did not show significant benefits for JPS, suggesting their primary mechanisms of action may not directly target the neural pathways responsible for conscious joint awareness. Regarding force sense, the meta‐analysis found no significant overall effect, a result heavily influenced by the very limited number of studies (*n* = 3) investigating this outcome. The isolated positive findings from Fu et al. [67] warrant further investigation. These results directly inform our clinical framework (Figure 16). For rehabilitating impaired joint position sense—a common clinical target—balance training and stroboscopic vision training are the recommended modalities with supporting evidence. The current evidence base is insufficient to make specific recommendations for force sense, highlighting an important avenue for future research.

### 4.5 Muscular Reaction Time

A delayed peroneal muscle reaction time is a key biomarker of impaired neuromuscular control in CAI, reflecting compromised reflexive stabilisation in response to sudden ankle perturbation [88, 89, 90, 91]. Our meta‐analysis indicates that exercise interventions collectively produce a dramatic and significant reduction in muscle reaction time (SMD •8.19), suggesting a potent effect on enhancing the speed of the neuromuscular feedback loop. This finding aligns with previous evidence highlighting the modifiability of this deficit through targeted training [92]. However, a critical constraint must be emphasised: the number of available studies was small (*n* = 3), and the included trials investigated different interventions (balance, neuromuscular and vibration training). This precluded a meaningful subgroup analysis to determine if any one modality is superior. Therefore, although the overall result is promising and underscores the general utility of exercise for improving reactive neuromuscular control, the specific mechanisms may vary—for example, by improving spinal‐level reflex excitability or central processing speed. Within our clinical decision framework (Figure 16), the implication is one of opportunity tempered by limited evidence. The significant overall effect justifies the inclusion of exercise as a primary strategy for addressing prolonged reaction time. However, the current evidence base is insufficient to recommend a specific intervention over others for this outcome alone. Consequently, the choice of modality for this deficit should be guided by the patient's other clinical presentations, leveraging the strong evidence for the selected intervention (e.g., balance training) on its other proven benefits. Future research prioritising this outcome is urgently needed to populate the framework with definitive recommendations.

### 4.6 Muscular Strength

Deficits in ankle muscular strength, particularly in the invertor and evertor muscle groups, are well‐documented in CAI and compromise the joint's active stabilisation capacity [93]. Our meta‐analysis yields a clear and clinically relevant distinction: exercise interventions are highly effective at improving concentric strength for both inversion (SMD 0.51) and eversion (SMD 0.49), but current evidence does not support a significant effect on eccentric strength. This divergent outcome likely stems from the fundamental specificity‐of‐training principle. The interventions studied, such as balance and strength training, predominantly incorporate concentric and isometric muscle actions, with limited emphasis on true eccentric loading. This highlights a critical gap in current rehabilitation paradigms, given the paramount importance of eccentric evertor strength in resisting inversion sprains. The efficacy for concentric strength was again intervention‐specific. Balance training, strength training and neuromuscular training significantly improved concentric inversion strength, whereas balance training and strength training were effective for concentric eversion strength. The repeated lack of significant strength gains from vibration training across multiple analyses suggests that the parameters commonly used (e.g., low‐frequency and whole‐body vibration) may be insufficient for robust morphological muscle adaptation, instead prioritising neuromuscular control [11, 40, 94]. Similarly, the nonsignificant findings for 3D training align with its focus on coordinated movement and rhythm rather than maximal force production. These findings critically inform the clinical decision framework (Figure 16). For the common clinical goal of improving concentric strength, strength training is the most direct and efficient choice, with balance training serving as a strong alternative that concurrently addresses other deficits. Conversely, the framework currently cannot recommend any specific exercise modality for eccentric strength deficits based on existing evidence, underscoring an urgent need for the development and evaluation of eccentric‐focused rehabilitation protocols in CAI.

### 4.7 Functional Performance

Functional performance tests, which assess integrated movements, such as jumping and hopping, provide a holistic measure of an individual's ability to perform sport‐like tasks under self‐generated loads [95]. Our meta‐analysis indicates that the effect of exercise interventions varies considerably depending on the metric used. For tests assessed by completion time, a large overall improvement was found (SMD −1.18). However, subgroup analysis revealed that this effect was almost entirely driven by the significant and substantial benefit of balance training. In contrast, strength training, 3D training and neuromuscular control training did not demonstrate significant effects. This suggests that the agility, coordination and postural control demanded by timed tests are most directly enhanced by the specific challenges inherent to balance training. For tests assessed by jump distance, a small but significant overall improvement was observed (SMD 0.38). The limited number of studies precluded subgroup analysis, but a review of individual trials suggests improvements may be associated with interventions, such as 3D training, that emphasise coordinated power generation [47]. The discrepancy between time and distance outcomes underscores that functional performance is multifaceted; improving speed and efficiency (time) may rely more on neuromuscular control, whereas improving power (distance) may have different drivers. When the rehabilitation goal is to improve functional performance in time‐based tasks (e.g., agility and shuttle runs), balance training is the intervention with the strongest and most consistent evidence. For goals centred on maximising jump distance, the current evidence supports the general use of exercise but is not yet robust enough to definitively prioritise one modality over another in the framework.

### 4.8 Strengths and Limitations

The findings must be interpreted within the context of certain limitations. The most significant constraint is the limited number of studies available for several intervention types (e.g., stroboscopic vision training and proprioceptive training) and specific outcome measures (e.g., force sense and eccentric strength). This compromised the statistical power of some subgroup analyses and precludes definitive conclusions for those areas. Secondly, the methodological quality of the included studies was variable, with a proportion exhibiting a moderate or high risk of bias, particularly concerning the blinding of participants and personnel, which is a common challenge in exercise intervention trials. Third, the presence of substantial statistical heterogeneity in some meta‐analyses indicates clinical or methodological diversity among the included studies, which was not fully explained by our subgroup analyses. Finally, this framework was derived from group‐level data and may not adequately account for individual patient factors influencing treatment response. Notably, its generalisability may be limited as most supporting trials enrolled young, athletic populations and some interventions require specialised equipment or supervision (e.g., stroboscopic training and vibration platforms), which affects clinical applicability. Moreover, real‐world effectiveness may be influenced by patient adherence and preference. Therefore, the framework is intended to serve as a practical guide rather than a rigid prescription.

Future research should prioritise large high‐quality RCTs for the underinvestigated interventions and outcomes highlighted herein. These studies should employ standardised clinically relevant outcome measures and detailed reporting of intervention parameters (e.g., dosage and intensity) to facilitate future synthesis. Exploring the effects of combined interventions and investigating moderators of treatment response (e.g., severity of instability and patient phenotype) will be essential for refining the proposed framework and advancing truly personalised rehabilitation for CAI.

## 5 Conclusion

This systematic review and meta‐analysis provided a definitive high‐resolution map of the differential effects of exercise interventions on the sensorimotor system in individuals with chronic ankle instability. The evidence robustly supports a paradigm shift from a generic prescription of exercise to a deficit‐targeted personalised approach. Among the modalities evaluated, balance training demonstrates the most comprehensive therapeutic profile, effectively addressing a broad range of impairments and thus serving as an optimal foundational intervention. However, the key clinical insight is that superior outcomes may be achieved by matching the patient's primary deficit to the most effective modality. The proposed evidence‐based framework synthesises these findings into a practical tool to guide clinical decision‐making. By empowering clinicians to select interventions based on the specific deficit profile of the individual with CAI, this research promises to enhance the precision, efficacy and efficiency of rehabilitation, ultimately improving functional outcomes and quality of life for this prevalent patient population.

## Author Contributions

**Jia Sheng Xu:** conceptualization, data curation, formal analysis, writing – original draft, writing – review and editing. **Hui Juan Lin:** conceptualization, data curation, formal analysis, writing – original draft, writing – review and editing. **Zhi Kun Li:** data curation, formal analysis, writing – original draft, writing – review and editing. **Zi Long Wang:** writing – original draft, writing – review and editing. **Chao Fan:** writing – original draft, writing – review and editing. **Hui Fang Chen:** writing – original draft, writing – review and editing. **Di Xie:** writing – original draft, writing – review and editing.

## Funding

This study was sponsored by Guangdong Basic and Applied Basic Research Foundation (Grant 2024A1515010442). The funders had no role in study design, data collection/analysis, decision to publish or preparation of the manuscript.

## Ethics Statement

The authors have nothing to report.

## Consent

The authors have nothing to report.

## Conflicts of Interest

The authors declare no conflicts of interest.

## Supporting information


Supporting Information S1

## Data Availability

All relevant data are included in the article or are available as online supplementary files.
