# Supplementary material for: Exercise Therapy for Chronic Ankle Instability: Which Modality for Which Deficit? A Systematic Review and Meta‐Analysis
Source: J Foot Ankle Res. 2026 Mar 2;19(1):e70142. doi: 10.1002/jfa2.70142 (PMC12953057; doi:10.1002/jfa2.70142)
Supplement: Supplementary file 1 — Supporting Information S1 [file JFA2-19-e70142-s001.docx]

**Supplemental Material**

**Exercise therapy for chronic ankle instability: which modality for which deficit? A systematic review and meta-analysis**

**Authors**

Jia Sheng. Xu^a,b,c,#^, BS; Hui Juan. Lin^a,b,c,#^, BS; Zhi Kun. Li^d^, BS; Zi Long. Wang^a^, BS; Chao. Fan^a^, BS; Hui Fang Chen^e,*^, MD; Di. Xie^a,b,c,*^, MD

**Affiliations**

^a^ University of Sports and Health, Guangzhou Sport University, Guangzhou, China.

^b^ Guangdong Provincial Key Laboratory of Intelligent Sports and Mental Health, China

^c^ Guangdong Key Laboratory of Human Sports Performance Science, Guangzhou,

510500, China

^d^ University of Xiamen, Xiamen, China.

^e^ University of Guangzhou Medical, Guangzhou, China

*Corresponding authors:

Hui Fang Chen

Email address: huifangcc@163.com

Institution: Guangzhou Medical University, Guangzhou 511436, Guangdong, China.

Di Xie

e-mail: 11452@gzsport.edu.cn

Guangzhou Sport University, Guangzhou 510500, Guangdong, China

**Contents**

[**Method S1. Full search strategy** 3](#_Toc220081338)

[**Table S1. Characteristic of included article** 7](#_Toc220081339)

[**Table S2.** **Egger's test results of included article** 31](#_Toc220081340)

[**Table S3.** **Quality rating of included article** 32](#_Toc220081341)

[**Table S4.** **Outcomes of certainty of evidence** 38](#_Toc220081342)

[**Figure S1.** **Publication bias of included articles** 49](#_Toc220081343)

[**Figure S2. Sensitive analysis of included articles** 51](#_Toc220081344)

[**Reference** 53](#_Toc220081345)

# **Method S1. Full search strategy**

***Search dates:*** Inception to July 1, 2025

***Search strategies for PubMed:***

(1) ankle instability-related terms:(“ankle instability” OR “chronic ankle instability” OR “functional ankle instability”)[Title/Abstract]

(2) exercise-related terms:(exercise or "Exercise intervention" or Exercises or "Physical Activity" or "Activities Physical" or "Activity Physical" or Training or "balance training" or "strength training" or "vibration training" or "Proprioception Training" or "Proprioceptive Training" or "Neuromuscular Control Training" or "Neuromuscular training" or Rehabilitation) [Title/Abstract]

(3) type of studies-related terms:(RCT OR “randomized controlled trial” OR “randomized controlled”)[All fields]

(4) Search strategies:(1) AND (2) AND (3)

***Search strategies for Proquest:***

(1) ankle instability-related terms:(“ankle instability” OR “chronic ankle instability” OR “functional ankle instability”)[All fields (excluding full text)]

(2) exercise-related terms:(exercise or "Exercise intervention" or Exercises or "Physical Activity" or "Activities Physical" or "Activity Physical" or Training or "balance training" or "strength training" or "vibration training" or "Proprioception Training" or "Proprioceptive Training" or "Neuromuscular Control Training" or "Neuromuscular training" or Rehabilitation) [All fields (excluding full text)]

(3) type of studies-related terms:(RCT OR “randomized controlled trial” OR “randomized controlled”)[All fields]

(4) Search strategies:(1) AND (2) AND (3)

***Search strategies for Web of Science:***

(1) ankle instability-related terms:(“ankle instability” OR “chronic ankle instability” OR “functional ankle instability”)[Topic]

(2) exercise-related terms:(exercise or "Exercise intervention" or Exercises or "Physical Activity" or "Activities Physical" or "Activity Physical" or Training or "balance training" or "strength training" or "vibration training" or "Proprioception Training" or "Proprioceptive Training" or "Neuromuscular Control Training" or "Neuromuscular training" or Rehabilitation) [Topic]

(3) type of studies-related terms:(RCT OR “randomized controlled trial” OR “randomized controlled”)[All fields]

(4) Search strategies:(1) AND (2) AND (3)

***Search strategies for Cochrane:***

(1) ankle instability-related terms:(“ankle instability” OR “chronic ankle instability” OR “functional ankle instability”)[Title/Keyword/Abstract]

(2) exercise-related terms:(exercise or "Exercise intervention" or Exercises or "Physical Activity" or "Activities Physical" or "Activity Physical" or Training or "balance training" or "strength training" or "vibration training" or "Proprioception Training" or "Proprioceptive Training" or "Neuromuscular Control Training" or "Neuromuscular training" or Rehabilitation) [Title/Keyword/Abstract]

(3) type of studies-related terms:(RCT OR “randomized controlled trial” OR “randomized controlled”)[All fields]

(4) Search strategies:(1) AND (2) AND (3)

***Search strategies for Embase:***

(1) ankle instability-related terms:(“ankle instability” OR “chronic ankle instability” OR “functional ankle instability”)[Title, Abstract or Author keywords]

(2) exercise-related terms:(exercise or "Exercise intervention" or Exercises or "Physical Activity" or "Activities Physical" or "Activity Physical" or Training or "balance training" or "strength training" or "vibration training" or "Proprioception Training" or "Proprioceptive Training" or "Neuromuscular Control Training" or "Neuromuscular training" or Rehabilitation) [Title, Abstract or Author keywords]

(3) type of studies-related terms:(RCT OR “randomized controlled trial” OR “randomized controlled”)[All fields]

(4) Search strategies:(1) AND (2) AND (3)

***Search strategies for Scopus:***

(1) ankle instability-related terms:(“ankle instability” OR “chronic ankle instability” OR “functional ankle instability”)[Article title, Abstract, Keywords]

(2) exercise-related terms:(exercise or "Exercise intervention" or Exercises or "Physical Activity" or "Activities Physical" or "Activity Physical" or Training or "balance training" or "strength training" or "vibration training" or "Proprioception Training" or "Proprioceptive Training" or "Neuromuscular Control Training" or "Neuromuscular training" or Rehabilitation) [Title, Abstract or Author keywords]

(3) type of studies-related terms:(RCT OR “randomized controlled trial” OR “randomized controlled”)[All fields]

(4) Search strategies:(1) AND (2) AND (3)

***Search strategies for CNKI:***

(1) ankle instability-related terms: (“ankle instability” OR “chronic ankle instability” OR “functional ankle instability”) [Title/Keyword/Abstract]

(2) exercise-related terms:(exercise or "Exercise intervention" or Exercises or "Physical Activity" or "Activities Physical" or "Activity Physical" or Training or "balance training" or "strength training" or "vibration training" or "Proprioception Training" or "Proprioceptive Training" or "Neuromuscular Control Training" or "Neuromuscular training" or Rehabilitation) [Title/Keyword/Abstract]

(3) type of studies-related terms:(RCT OR “randomized controlled trial” OR “randomized controlled”) [All fields]

(4) Search strategies:(1) AND (2) AND (3)

***Search strategies for Wanfang:***

All fields: (exercise OR training OR exercise intervention OR exercise therapy OR balance training OR proprioception training OR vibration training OR strength training OR neuromuscular training OR neuromuscular control training OR rehabilitation) AND (chronic ankle instability OR ankle instability OR functional ankle instability) AND (randomized controlled trial OR random OR RCT)

***Search strategies for VIP:***

((((((((((Any Field = exercise OR Any Field = training) OR Any Field = exercise intervention) OR Any Field = exercise therapy) OR Any Field = balance training) OR Any Field = proprioception training) OR Any Field = vibration training) OR Any Field = strength training) OR Any Field = neuromuscular training) OR Any Field = neuromuscular control training) OR Any Field = rehabilitation) AND ((Any Field = chronic ankle instability OR Any Field = ankle instability) OR Any Field = functional ankle instability)) AND ((Any Field = randomized controlled trial OR Any Field = random) OR Any Field = RCT))

#

# **Table S1. Characteristic of included article: A. Pain; B. Patient-reported functional impairment and stability; C. Dynamic balance; D. Joint position sense; E.** **Force sense; F. Muscular reaction time; G.** **Concentric inversion muscular strength; H. Eccentric inversion muscular strength; I.** **Concentric eversion muscular strength; J.** **Eccentric eversion muscular strength; K. Functional performance (based on the time completing the test); L. Functional performance (based on the distance completing the test))**

| **A.** | | | | | | |  |  |
| --- | --- | --- | --- | --- | --- | --- | --- | --- |
| **First Author** | **Year** | **Category** | **Sample Size*** | **Mean Age (y) (mean±SD)** | **Outcomes** | **Significance** | **Funding source** | ***Author COI*** |
| Park et al (1)^1^ | 2023 | Strength training | 7:7 | E:(22.7±3.6)  C: (21.7±2.2) | VAS | YES | No external funding | No conflict of interest |
| Park et al (2)^1^ | 2023 | Proprioceptive training | 7:7 | E:(22.7±3.6)  C: (21.7±2.2) | VAS | YES | No external funding | No conflict of interest |
| Yang et al^2^ | 2022 | 3D training | 12:12 | E:(22.5±3.1)  C: (22.3±2.2) | VAS | NO | No external funding | No conflict of interest |

| **B．** |  |  |  |  |  |  |  |  |
| --- | --- | --- | --- | --- | --- | --- | --- | --- |
| **First Author** | **Year** | **Category** | **Sample Size*** | **Mean Age (y) (mean±SD)** | **Outcomes** | **Significance** | **Funding source** | ***Author COI*** |
| Tang et al^3^ | 2017 | Vibration training | 15:15 | E:(21.90±2.51) C:( 22.00±1.94) | CAIT | NO | No external funding | No conflict of interest |
| Liu et al^4^ | 2019 | Vibration training | 19:15 | Not applicable | AJFAT | NO | None declared | None declared |
| Fan et al^5^ | 2022 | Balance training | 15/7:13/8 | E:(21.36±2.17) C:( 22.19±2.18) | CAIT | YES | No external funding | No conflict of interest |
| Wang et al^6^ | 2023 | Balance training | 14/10:15/0 | E:(20.46±1.02) C:( 20.33±0.86) | CAIT | YES | Funding Agency | No conflict of interest |
| Kim et al (2)^7^ | 2021 | Balance training | 12/13:13/11 | E:(29.76±10.01) C:(29.6±9.41) | CAIT | YES | No external funding | No conflict of interest |
| Parlakidis et al^8^ | 2024 | Balance training | 6/4:9/1 | E:(21.4±1.95) C:(23.7±1.63) | CAIT | NO | No external funding | No conflict of interest |
| Cui et al^9^ | 2022 | Balance training | 11/9:12/8 | E:(34.50±6.09) C:(35.65±5.74) | CAIT | NO | Funding Agency | None declared |
| Sun et al (2)^10^ | 2023 | Balance training | 12/0:12/0 | E:(21.33±1.30) C:(21.25±1.71) | CAIT | YES | No external funding | No conflict of interest |
| Park et al (2)^11^ | 2024 | Balance training | 17/0:17/0 | E:(14.1±0.7) C:(14.4±0.7) | CAIT | YES | No external funding | No conflict of interest |
| Kim et al (2)^12^ | 2022 | Strength training | 22:22 | E:(29.5±10.7) C:(27.4±8.3) | CAIT | NO | Funding Agency | No conflict of interest |
| Luo et al^13^ | 2017 | Strength training | 10/10:11/9 | E:(28.46±5.00) C:(28.53±5.83) | CAIT | YES | Funding Agency | None declared |
| Zhang et al^14^ | 2021 | Strength training | 11/19:13/17 | E:(20.3±3.8) C:(18.4±4.0) | CAIT | YES | No external funding | No conflict of interest |
| Park et al (1)^1^ | 2023 | Strength training | 7:7 | E:(22.7±3.6) C:(21.7±2.2) | CAIT | YES | No external funding | No conflict of interest |
| Zhou et al^15^ | 2021 | Strength training | 13/5:12/4 | E:(20.78±2.05) C:(22.25±2.44) | CAIT | NO | No external funding | No conflict of interest |
| Zhou et al^16^ | 2018 | Strength training | 16/17:17/16 | E:(31.76±7.05) C:(33.09士7.75) | CAIT | YES | No external funding | No conflict of interest |
| Park et al (1)^11^ | 2024 | Strength training | 17/0:17/0 | E:(14.1±0.7) C:(14.4±0.7) | CAIT | NO | No external funding | No conflict of interest |
| Li et al^17^ | 2023 | 3D training | 15:15 | E:(20.67±2.2) C:(21.47±2.0) | CAIT | NO | No external funding | None declared |
| Li et al^18^ | 2024 | 3D training | 10/13:12/13 | E:(19.86±1.46) C:(19.89±1.76) | CAIT | NO | Funding Agency | None declared |
| Wang et al^19^ | 2019 | 3D training | 12:12 | Not applicable | CAIT | YES | No external funding | None declared |
| Yang et al^2^ | 2022 | 3D training | 12:12 | E:(21.4±3.90) C:(22.33±2.15) | AJFAT | NO | No external funding | No conflict of interest |
| Naderi et al^20^ | 2025 | 3D training | 13/8:14/8 | E:(35.5±9.7) C:(30.9±9.5) | CAIT | YES | No external funding | None declared |
| Xu et al^21^ | 2024 | 3D training | 9/8:10/7 | E:(21.8±2.0) C:(23.0±2.5) | CAIT | YES | Funding Agency | No conflict of interest |
| Yin et al^22^ | 2023 | Neuromuscular control training | 9/8:10/7 | E:(20.3±1.8 ) C:(20.4±1.7) | AJFAT | YES | Funding Agency | None declared |
| Kim et al (1)^12^ | 2022 | Neuromuscular control training | 22:22 | E:(27.1±5.8) C:(22.33±2.15) | CAIT | NO | Funding Agency | No conflict of interest |
| Lbrahim et al^23^ | 2020 | Neuromuscular control training | 20:20 | E:(19.15±1.66) C:(19.6±1.5) | AJFAT | YES | No external funding | None declared |
| Liu et al^24^ | 2022 | Neuromuscular control training | Not applicable | E:(20.31±2.02) C:(20.56±2.00) | CAIT | YES | No external funding | No conflict of interest |
| Yuan et al^25^ | 2023 | Neuromuscular control training | 12/4:12/4 | E:(27.38±7.38)  C:(29.67±9.41) | CAIT | YES | No external funding | No conflict of interest |
| Kim et al (1)^7^ | 2021 | Stroboscopic vision training | 17/7:13/11 | E:(21.58±1.78) C:(21.25±1.71) | CAIT | YES | No external funding | No conflict of interest |
| Sun et al (1)^10^ | 2023 | Stroboscopic vision training | 12:12 | E:(21.73±1.67) C:(21.10±2.18) | CAIT | NO | No external funding | No conflict of interest |
| Wu et al^26^ | 2024 | Stroboscopic vision training | 6/5:7/3 | E:(22.7±2.1) C:(21.7±2.2) | CAIT | YES | Funding Agency | No conflict of interest |
| Park et al (2)^1^ | 2023 | Propioceptive training | 7:7 | E:(21.90±2.51) C:(22.00±1.94) | CAIT | YES | No external funding | No conflict of interest |

| **C.** | | | | | | |  |  |
| --- | --- | --- | --- | --- | --- | --- | --- | --- |
| **First Author** | **Year** | **Category** | **Sample Size*** | **Mean Age (y) (mean±SD)** | **Outcomes** | **Significance** | **Funding source** | ***Author COI*** |
| Cloak et al^27^ | 2010 | Vibration training | 0/19 :0/19 | E:(19±0.8)  C:(19±1.3) | SEBT | YES | No external funding | None declared |
| Astorino et al (1)^28^ | 2021 | Vibration training | 5/7 :4/8 | E:(20.06±0.94) C:(20.83±1.58) | mSEBT | YES | Funding Agency | None declared |
| Shamseddini et al^29^ | 2021 | Vibration training | 4/8 :5/5 | E:(35.83±12.08) C:(38.30±10.49) | mSEBT | NO | Funding Agency | No conflict of interest |
| Sierra-Guzm et al (1)^30^ | 2018 | Vibration training | 11/6 :12/5 | E:(22.4±2.6) C:(23.6±3.4) | mSEBT | YES | No external funding | None declared |
| Chang et al (1)^31^ | 2021 | Vibration training | 0/21 :0/21 | E:(20.31±1.28) C:(21.23±1.47) | SEBT | YES | No external funding | No conflict of interest |
| Cain et al^32^ | 2017 | Balance training | 4/7 :7/4 | E:(16.45±0.93) C:(16.55±1.29) | mSEBT | YES | No external funding | None declared |
| Cain et al (1)^33^ | 2020 | Balance training | 8/2 :4/7 | E:(16.40±0.97) C:(16.45±1.04) | mSEBT | NO | Funding Agency | No conflict of interest |
| Wang et al^6^ | 2023 | Balance training | 14/10 :15/0 | E:(20.46±1.02) C:(20.33±0.86) | mSEBT | YES | Funding Agency | No conflict of interest |
| Kim et al (1)^7^ | 2021 | Balance training | 12/13 :13/11 | E:(29.76±10.009) C:(29.67±9.407) | mSEBT | NO | No external funding | No conflict of interest |
| Linens et al^34^ | 2016 | Balance training | 17:17 | E:(22.94±2.77) C:(23.18±3.64) | mSEBT | YES | Funding Agency | No conflict of interest |
| Mckeon et al^35^ | 2008 | Balance training | 6/10 :6/9 | E:(22.2±4.5) C:(19.5±1.2) | mSEBT | YES | Funding Agency | No conflict of interest |
| Parlakidis et al^8^ | 2024 | Balance training | 6/4 :9/1 | E:(21.4±1.95) C:(23.7±1.63) | mSEBT | YES | No external funding | No conflict of interest |
| Sierra-Guzm et al (2)^30^ | 2018 | Balance training | 10/6 :12/5 | E:(21.8±2.1) C:(23.6±3.4) | mSEBT | NO | No external funding | None declared |
| Chang et al (2)^31^ | 2021 | Balance training | 0/21 :0/21 | E:(20.43±1.25) C:(21.23±1.47) | SEBT | YES | No external funding | No conflict of interest |
| Taghavi et al^36^ | 2022 | Balance training | 7:7 | E:(23.14±1.34 ) C:(22.42±2.43) | mSEBT | YES | Funding Agency | No conflict of interest |
| Cui et al^9^ | 2022 | Balance training | 11/9 :12/8 | E:(34.50±6.09) C:(35.65±5.74) | mSEBT | NO | Funding Agency | None declared |
| Sun et al (1)^10^ | 2023 | Balance training | 12/0:12/0 | E:(21.33±1.30) C:(21.25±1.71) | mSEBT | YES | No external funding | No conflict of interest |
| Park et al (2)^11^ | 2024 | Balance training | 17/0:17/0 | E:(14.1±0.7) C:( 14.4±0.7) | mSEBT | NO | No external funding | No conflict of interest |
| Reyes et al^37^ | 2024 | Balance training | 4/11 :8/7 | E:(22.7±3.3) C:( 22.3±3.1) | mSEBT | NO | Funding Agency | No conflict of interest |
| Cain et al (2)^33^ | 2020 | Strength training | 5/7 :4/7 | E:(16.42±1.00) C:(16.45±1.04) | mSEBT | NO | Funding Agency | No conflict of interest |
| Curz-D et al^38^ | 2020 | Strength training | 11/15 :12/14 | E:(35.4±10.46) C:(36.3±11.98) | mSEBT | NO | No external funding | None declared |
| Hall et al (1)^39^ | 2015 | Strength training | 5/8 :7/6 | E:(19.7±2.2) C:(20.5±2.1) | mSEBT | NO | No external funding | None declared |
| Cruz-Diaz et al^40^ | 2014 | Strength training | 15/20 :20/15 | E:(31.89±10.52) C:( 28.83±7.91) | mSEBT | YES | No external funding | No conflict of interest |
| Kim et al (1)^12^ | 2022 | Strength training | 22:22 | E:(29.5±10.7) C:(27.4±8.3) | mSEBT | YES | Funding Agency | No conflict of interest |
| Luo et al^13^ | 2017 | Strength training | 10/10 :11/9 | E:(28.46±5.00) C:(28.53±5.83) | SEBT | YES | Funding Agency | None declared |
| Zhang et al^14^ | 2021 | Strength training | 11/19 :13/17 | E:(20.3±3.8) C:(18.4±4.0) | SEBT | YES | No external funding | No conflict of interest |
| Melam et al^41^ | 2018 | Strength training | 15:15 | E:(21.0±2.2) C:(21.3±2.3) | mSEBT | NO | No external funding | None declared |
| Park et al (1)^1^ | 2023 | Strength training | 7:7 | E:(22.7±3.6) C:(21.7±2.2) | mSEBT | YES | No external funding | No conflict of interest |
| Smith et al^42^ | 2018 | Strength training | 6/7 6/7 | E:(20.1±1.69) C:(20.9±1.26) | mSEBT | YES | No external funding | No conflict of interest |
| Zhou et al^15^ | 2021 | Strength training | 13/5 :12/4 | E:(20.78±2.05) C:(22.25±2.44) | mSEBT | NO | No external funding | No conflict of interest |
| Zhou et al^16^ | 2018 | Strength training | 16/17 :17/16 | E:(31.76±7.05) C:( 33.09 ±7.75) | SEBT | YES | No external funding | No conflict of interest |
| Park et al (1)^11^ | 2024 | Strength training | 17/0:17/0 | E:(14.1±0.7) C:( 14.4±0.7) | mSEBT | YES | No external funding | No conflict of interest |
| Cruz-D et al^43^ | 2020 | 3D training | 11/15 :12/14 | E:(35.4±10.46) C:(36.3±11.98) | mSEBT | YES | No external funding | No conflict of interest |
| Jiang et al^44^ | 2022 | 3D training | 26:25 | E:(21.8±1.6) C:(22.5±1.5) | mSEBT | NO | No external funding | No conflict of interest |
| Yang et al^2^ | 2022 | 3D training | 12:12 | E:(21.42±3.90 )  C:(22.33±2.15) | mSEBT | YES | No external funding | No conflict of interest |
| Naderi et al^20^ | 2025 | 3D training | 13/8 :14/8 | E:(35.5±9.7) C:(30.9±9.5) | mSEBT | YES | No external funding | None declared |
| Xu et al^21^ | 2024 | 3D training | 9/8 :10/7 | E:(21.8±2.0) C:(23.0±2.5) | mSEBT | YES | Funding Agency | No conflict of interest |
| Li et al^18^ | 2024 | 3D training | 10/13 :12/13 | E:(19.86±1.46) C:(19.89±1.76) | mSEBT | NO | Funding Agency | None declared |
| Liu et al^45^ | 2024 | 3D training | 15:15 | E:(20.73±1.22) C:(20.80±1.32) | mSEBT | NO | No external funding | No conflict of interest |
| Hall et al (2)^39^ | 2015 | Neuromuscular control training | 5/8 :7/6 | E:(18.9±1.3) C:(20.5±2.1) | mSEBT | NO | No external funding | None declared |
| Yin et al^22^ | 2023 | Neuromuscular control training | 9/8 :10/7 | E:(20.3±1.8 ) C:(20.4±1.7) | SEBT | YES | Funding Agency | None declared |
| Kim et al (2)^12^ | 2022 | Neuromuscular control training | 22:22 | E:(27.1±5.8) C:(22.33±2.15) | mSEBT | YES | Funding Agency | No conflict of interest |
| Liu et al^24^ | 2022 | Neuromuscular control training | 7/8 :7/8 | Not applicable | mSEBT | NO | No external funding | No conflict of interest |
| Shih et al^46^ | 2018 | Neuromuscular control training | 12/4 :12/4 | E:(26.9±5.8) C:(27.9±6.6) | mSEBT | NO | Funding Agency | None declared |
| Yuan et al^25^ | 2023 | Neuromuscular control training | 17/7 :13/11 | E:(20.31±2.02) C:( 20.56±2.00) | mSEBT | YES | No external funding | No conflict of interest |
| Kim et al (2)^7^ | 2021 | Stroboscopic vision training | 12/0:12/0 | E:( 27.38±7.383) C:(29.67±9.407) | mSEBT | YES | No external funding | No conflict of interest |
| Sun et al (2)^10^ | 2023 | Stroboscopic vision training | 6/5 :7/3 | E:(21.58±1.78) C:(21.25±1.71) | mSEBT | YES | No external funding | No conflict of interest |
| Wu et al^26^ | 2024 | Stroboscopic vision training | 6/6 :4/8 | E:(21.73±1.67) C:(21.10±2.18) | mSEBT | YES | Funding Agency | No conflict of interest |
| Astorino et al (2)^28^ | 2021 | Proprioceptive training | 7:7 | E:(19.66±3.28) C:(20.83±1.58) | mSEBT | NO | Funding Agency | None declared |
| Park et al (2)^1^ | 2023 | Proprioceptive training | 0/10 :0/10 | E:(22.7±2.1) C:(21.7±2.2) | mSEBT | YES | No external funding | No conflict of interest |
| Liang et al^47^ | 2019 | Proprioceptive training | 15:15 | E:(21.56±2.76) C:(21.00±2.98) | mSEBT | NO | No external funding | No conflict of interest |
| Bagherian et al^48^ | 2019 | Other | 0/19 :0/19 | E:(21.1±1.7) C:(20.9±1.8) | mSEBT | YES | No external funding | No conflict of interest |

| **D.** | | | | | | |  |  |
| --- | --- | --- | --- | --- | --- | --- | --- | --- |
| **First Author** | **Year** | **Category** | **Sample Size*** | **Mean Age (y) (mean±SD)** | **Outcomes** | **Significance** | **Funding source** | ***Author COI*** |
| Tang et al^3^ | 2017 | Vibration training | 15:15 | E:(21.90±2.51) C:(22.00±1.94) | Half of the maximum inversion angle | NO | No external funding | No conflict of interest |
| Shamseddini et al^29^ | 2021 | Vibration training | 19:15 | E:(35.83±12.08) C:(38.30±10.49) | Active and passive inversion at 5°and 15° | NO | Funding Agency | No conflict of interest |
| Chang et al (1)^31^ | 2021 | Vibration training | 15/7:13/8 | E:(20.31±1.28) C:(21.23±1.47) | Active and passive inversion at 5°and 15° | NO | No external funding | No conflict of interest |
| Bernier et al^49^ | 1998 | Balance training | 14/10:15/0 | 22.53 ± 3.95 | Active and passive inversion at 15°and maximum inversion angle minus 5° | NO | No external funding | None declared |
| Hua et al^50^ | 2017 | Balance training | 12/13:13/11 | E:(20.6±1.37) C:(20.9±1.03) | Active and passive inversion at 40° | YES | Funding Agency | None declared |
| Wang et al^6^ | 2023 | Balance training | 6/4:9/1 | E:(20.46±1.02) C:(20.33±0.86) | Active inversion repositioning at 30° | NO | Funding Agency | No conflict of interest |
| Jain et al^51^ | 2014 | Balance training | 11/9:12/8 | E:(21.90±2.51) C:(22.00±1.94) | Passive repositioning inversion at 15° and 30° | YES | No external funding | No conflict of interest |
| Chang et al (2)^31^ | 2021 | Balance training | 12/0:12/0 | E:(20.43±1.25) C:(21.23±1.47) | Active and passive inversion at 5°and 15° | NO | No external funding | No conflict of interest |
| Liu et al^45^ | 2024 | 3D training | 17/0:17/0 | E:(20.73±1.22) C:(20.80±1.32) | Active inversion at 15° | NO | No external funding | No conflict of interest |
| Wu et al^26^ | 2024 | Stroboscopic vision training | 22:22 | E:(21.73±1.67) C:(21.10±2.18) | Active repositioning inversion at 10°, 12°, 14°, and 16° | YES | Funding Agency | No conflict of interest |
| Bagherian et al^48^ | 2019 | Other | 10/10:11/9 | E:(21.1±1.7) C:(20.9±1.8) | Active repositioning inversion at 15° and calculating the mean of three absolute error values. | YES | No external funding | No conflict of interest |

| **E.** | | | | | | |  |  |
| --- | --- | --- | --- | --- | --- | --- | --- | --- |
| **First Author** | **Year** | **Category** | **Sample Size*** | **Mean Age (y) (mean±SD)** | **Outcomes** | **Significance** | **Funding source** | ***Author COI*** |
| Wang et al^19^ | 2019 | 3D training | 12:12 | Not applicable | Not applicable | YES | No external funding | None declared |
| Fu et al^52^ | 2020 | Balance training | 10:10 | Not applicable | 25% of maximum isometric strength | YES | No external funding | No conflict of interest |
| Smith et al^53^ | 2012 | Strength training | 10/10:10/10 | E:(20.9±2.2) C:(20.2±2.1) | 20% and 30% of maximum isometric strength | NO | No external funding | None declared |

| **F**. | | | | | | |  |  |
| --- | --- | --- | --- | --- | --- | --- | --- | --- |
| **First Author** | **Year** | **Category** | **Sample Size*** | **Mean Age (y) (mean±SD)** | **Outcomes** | **Significance** | **Funding source** | ***Author COI*** |
| Liu et al^4^ | 2019 | Vibration training | 19:15 | Not applicable | Peroneus longus muscular reaction time | YES | None declared | None declared |
| Wang et al^6^ | 2023 | Balance training | 14/10:0/15 | E:(20.46±1.02) C:(20.33±0.86) | Peroneus longus muscular reaction time | YES | Funding Agency | No conflict of interest |
| Yuan et al^25^ | 2023 | Neuromuscular training | 12/4:12/4 | E:(20.31±2.02) C:(20.56±2.00) | Peroneus longus muscular reaction time | YES | No external funding | No conflict of interest |

| **G**. | | | | | | |  |  |
| --- | --- | --- | --- | --- | --- | --- | --- | --- |
| **First Author** | **Year** | **Category** | **Sample Size*** | **Mean Age (y) (mean±SD)** | **Outcomes** | **Significance** | **Funding source** | ***Author COI*** |
| Tang et al^3^ | 2017 | Vibration training | 15/0:15/0 | E:(21.90±2.51) C:(22.00±1.94) | Concentric inversion muscle strength at 60°/s | NO | No external funding | No conflict of interest |
| Kim et al^54^ | 2019 | Vibration training | 10:10 | E:(21.60±1.65) C:(22.10±1.45) | Concentric inversion muscle strength at 60°/s and 120°/s | NO | No external funding | None declared |
| Shamseddini et al^29^ | 2021 | Vibration training | 8:5 | E:(35.8±12.1)  C:(38.3±10.5) | Concentric inversion muscle strength at 90°/s | NO | Funding Agency | No conflict of interest |
| Chang et al (1)^31^ | 2021 | Vibration training | 0/21:0/21 | E:(20.31±1.28) C:(21.23±1.47) | Concentric inversion muscle strength | NO | No external funding | No conflict of interest |
| Deussen et al (1)^55^ | 2018 | Balance training | 3/4:4/2 | E:(30.0±6.83) C:(26.67±6.22) | Concentric inversion muscle strength at 60°/s and 180°/s | NO | No external funding | No conflict of interest |
| Fu et al^52^ | 2020 | Balance training | 10:10 | Not applicable | Concentric inversion muscle strength at 60°/s | YES | No external funding | No conflict of interest |
| Wang et al^6^ | 2023 | Balance training | 14/10:15/0 | E:(20.46±1.02) C:(20.33±0.86) | Concentric inversion muscle strength at 30°/s and 120°/s | YES | Funding Agency | No conflict of interest |
| Chang et al (2)^31^ | 2021 | Balance training | 0/21:0/21 | E:(20.43±1.25) C:(21.23±1.47) | Concentric inversion muscle strength | NO | No external funding | No conflict of interest |
| Zhao et al^56^ | 2022 | Balance training | 8/0:8/0 | E:(16.75±1.28) C:(17.00±1.07) | Concentric inversion muscle strength | YES | No external funding | No conflict of interest |
| Hall et al (1)^39^ | 2015 | Strength training | 5/8:7/6 | E:(19.7±2.2) C:(20.5±2.1) | Concentric inversion muscle strength at 60°/s | NO | No external funding | None declared |
| Smith et al^53^ | 2012 | Strength training | 10/10:10/10 | E:(20.9±2.2) C:(20.2±2.1) | Concentric inversion muscle strength at 30°/s | YES | No external funding | None declared |
| Jiang et al^44^ | 2022 | 3D training | 26:25 | E:(21.8±1.6) C:(22.5±1.5) | Concentric inversion muscle strength at 60°/s and 180°/s | NO | No external funding | No conflict of interest |
| Xu et al^21^ | 2024 | 3D training | 9/8:10/7 | E:(21.8±2.0) C:(23.0±2.5) | Concentric inversion muscle strength | NO | Funding Agency | No conflict of interest |
| Liu et al^45^ | 2024 | 3D training | 15:15 | E:(20.73±1.22) C:(20.80±1.32) | Concentric inversion muscle strength | YES | No external funding | No conflict of interest |
| Hall et al (2)^39^ | 2015 | Neuromuscular control training | 5/8:7/6 | E:(18.9±1.3) C:(20.5±2.1) | Concentric inversion muscle strength at 60°/s and 180°/s | YES | No external funding | None declared |
| Yuan et al^25^ | 2023 | Neuromuscular control training | 12/4:12/4 | E:(20.3±2.0) C:( 20.6±2.0) | Concentric inversion muscle strength at 60°/s and 180°/s | YES | No external funding | No conflict of interest |
| Liang et al^47^ | 2019 | Proprioceptive training | 0/10:0/10 | E:(21.56±2.76) C:(21.00±2.98) | Concentric inversion muscle strength at 60°/s | YES | No external funding | No conflict of interest |
| Liang et al^57^ | 2022 | Proprioceptive training | 0/15:0/15 | E:(21.56±2.76) C:(21±2.98) | Concentric inversion muscle strength | NO | No external funding | None declared |
| Bagherian et al^48^ | 2019 | Other | 20:20 | E:(21.1±1.7) C:(20.9±1.8) | Concentric inversion muscle strength at 60°/s | NO | No external funding | No conflict of interest |
| Deussen et al (2)^55^ | 2018 | Other | 6/0:4/2 | E:(29.83±8.18) C:(26.67±6.22) | Concentric inversion muscle strength at 60°/s and 120°/s | NO | No external funding | No conflict of interest |

| **H**. | | | | | | |  |  |
| --- | --- | --- | --- | --- | --- | --- | --- | --- |
| **First Author** | **Year** | **Category** | **Sample Size*** | **Mean Age (y) (mean±SD)** | **Outcomes** | **Significance** | **Funding source** | ***Author COI*** |
| Shamseddini et al^29^ | 2021 | Vibration training | 8:5 | E:(35.83±12.08) C:(38.30±10.49) | Eccentric inversion muscle strength at 90°/s | NO | Funding Agency | No conflict of interest |
| Chang et al (2)^31^ | 2021 | Vibration training | 0/21:0/21 | E:(20.31±1.28) C:(21.23±1.47) | Eccentric inversion muscle strength at 30°/s and 120°/s | NO | No external funding | No conflict of interest |
| Fan et al^5^ | 2022 | Balance training | 15/7:13/8 | E:(21.36±2.17) C:(22.19±2.18) | Eccentric inversion muscle strength at 90°/s and 180°/s | NO | No external funding | No conflict of interest |
| Chang et al (1)^31^ | 2021 | Balance training | 0/21:0/21 | E:(20.43±1.25) C:(21.23±1.47) | Eccentric inversion muscle strength at 30°/s and 120°/s | YES | No external funding | No conflict of interest |

| **I.** | | | | | | |  |  |
| --- | --- | --- | --- | --- | --- | --- | --- | --- |
| **First Author** | **Year** | **Category** | **Sample Size*** | **Mean Age (y) (mean±SD)** | **Outcomes** | **Significance** | **Funding source** | ***Author COI*** |
| Tang et al^3^ | 2017 | Vibration training | 15/0:15/0 | E:(21.90±2.51) C:(22.00±1.94) | Concentric eversion muscle strength at 60°/s | NO | Funding Agency | No conflict of interest |
| Kim et al^54^ | 2019 | Vibration training | 10:10 | E:(21.60±1.65) C:(22.10±1.45) | Concentric eversion muscle strength at 60°/s and 120°/s | NO | No external funding | None declared |
| Shamseddini et al^29^ | 2021 | Vibration training | 8:5 | E:(35.83±12.08) C:(38.30±10.49) | Concentric eversion muscle strength at 90°/s | NO | Funding Agency | No conflict of interest |
| Sierra-Guzm et al (1)^30^ | 2018 | Vibration training | 11/6:12/5 | E:(22.4±2.6) C:(23.6±3.4) | Concentric eversion muscle strength at 60°/s，180°/s and 300°/s | NO | No external funding | None declared |
| Chang et al (1)^31^ | 2021 | Vibration training | 0/21:0/21 | E:(20.31±1.28) C:(21.23±1.47) | Concentric eversion muscle strength at 30°/s and 120°/s | YES | No external funding | No conflict of interest |
| Deussen et al (1)^55^ | 2018 | Balance training | 3/4:4/2 | E:(30.0±6.83) C:(26.67±6.22) | Concentric eversion muscle strength | NO | No external funding | No conflict of interest |
| Fu et al^52^ | 2020 | Balance training | 10:10 | Not applicable | Concentric eversion muscle strength at 60°/s and 180°/s | YES | No external funding | No conflict of interest |
| Wang et al^6^ | 2023 | Balance training | 14/10:15/0 | E:(20.46±1.02) C:(20.33±0.86) | Concentric eversion muscle strength at 60°/s | YES | Funding Agency | No conflict of interest |
| Sierra-Guzm et al (2)^30^ | 2018 | Balance training | 10/6F:12/5F | E:(21.8±2.1) C:(23.6±3.4) | Concentric eversion muscle strength at 60°/s，180°/s and 300°/s | NO | No external funding | None declared |
| Chang et al (2)^31^ | 2021 | Balance training | 0/21:0/21 | E:(20.43±1.25) C:(21.23±1.47) | Concentric eversion muscle strength at 30°/s and 120°/s | YES | No external funding | No conflict of interest |
| Zhao et al^56^ | 2022 | Balance training | 8/0:8/0 | E:(16.75±1.28) C:(17.00±1.07) | Concentric eversion muscle strength at 60°/s | YES | No external funding | No conflict of interest |
| Hall et al (1)^39^ | 2015 | Strength training | 5/8:7/6 | E:(19.7±2.2) C:(20.5±2.1) | Concentric eversion muscle strength muscle strength | NO | No external funding | None declared |
| Smith et al^53^ | 2012 | Strength training | 10/10:10/10 | E:(20.9±2.2) C:(20.2±2.1) | Concentric eversion muscle strength | YES | No external funding | None declared |
| Jiang et al^44^ | 2022 | 3D training | 26:25 | E:(21.8±1.6) C:(22.5±1.5) | Concentric eversion muscle strength at 60°/s | NO | No external funding | No conflict of interest |
| Xu et al^21^ | 2024 | 3D training | 9/8:10/7 | E(21.8±2.0) C:(23.0±2.5) | Concentric eversion muscle strength at 30°/s | YES | Funding Agency | No conflict of interest |
| Liu et al^45^ | 2024 | 3D training | 15:15 | E:(20.73±1.22) C:(20.80±1.32) | Concentric eversion muscle strength at 60°/s and 180°/s | NO | No external funding | No conflict of interest |
| Hall et al (2)^39^ | 2015 | Neuromuscular control training | 5/8:7/6 | E:(18.9±1.3) C:(20.5±2.1) | Concentric eversion muscle strength muscle strength | NO | No external funding | None declared |
| Yuan et al^25^ | 2023 | Neuromuscular control training | 12/4:12/4 | E:(20.31±2.02) C:( 20.56±2.00) | Concentric eversion muscle strength | YES | No external funding | No conflict of interest |
| Liang et al^47^ | 2019 | Propioceptive training | 0/10:0/10 | E:(21.56±2.76) C:(21.00±2.98) | Concentric eversion muscle strength at 60°/s and 180°/s | NO | No external funding | No conflict of interest |
| Liang et al^57^ | 2022 | Propioceptive training | 0/15:0/15 | E:(21.56±2.76) C:(21±2.98) | Concentric eversion muscle strength at 60°/s and 180°/s | YES | No external funding | None declared |
| Bagherian et al^48^ | 2019 | Other | 20:20 | E:(21.1±1.7) C:(20.9±1.8) | Concentric eversion muscle strength at 60°/s | YES | No external funding | No conflict of interest |
| Deussen et al (2)^55^ | 2018 | Other | 6/0:4/2 | E:(29.83±8.18) C:(26.67±6.22) | Concentric eversion muscle strength | NO | No external funding | No conflict of interest |

| **J.** | | | | | | |  |  |
| --- | --- | --- | --- | --- | --- | --- | --- | --- |
| **First Author** | **Year** | **Category** | **Sample Size*** | **Mean Age (y) (mean±SD)** | **Outcomes** | **Significance** | **Funding source** | ***Author COI*** |
| Shamseddini et al^29^ | 2021 | Vibration training | 8:5 | E:(35.83±12.08) C:(38.30±10.49) | Eccentric version muscle strength at 90°/s | NO | Funding Agency | No conflict of interest |
| Sierra-Guzm et al (2)^30^ | 2018 | Vibration training | 11/6:12/5 | E:(22.4±2.6) C:(23.6±3.4) | Eccentric eversion muscle strength at 60°/s，180°/s and 300°/s | NO | No external funding | None declared |
| Chang et al (2)^31^ | 2021 | Vibration training | 0/21:0/21 | E:(20.31±1.28) C:(21.23±1.47) | Eccentric eversion muscle strength at 30°/s and 120°/s | YES | No external funding | No conflict of interest |
| Fan et al^5^ | 2022 | Balance training | 15/7:13/8 | E:(21.36±2.17) C:(22.19±2.18) | Eccentric inversion muscle strength at 90°/s and 180°/s | NO | No external funding | No conflict of interest |
| Sierra-Guzm et al (1)^30^ | 2018 | Balance training | 10/6:12/5 | E:(21.8±2.1) C:(23.6±3.4) | Eccentric eversion muscle strength at 60°/s，180°/s and 300°/s | NO | No external funding | None declared |
| Chang et al (1)^31^ | 2021 | Balance training | 0/21:0/21 | E:(20.43±1.25) C:(21.23±1.47) | Eccentric eversion muscle strength at 60°/s，180°/s and 300°/s | YES | No external funding | No conflict of interest |

| **K**. | | | | | | |  |  |
| --- | --- | --- | --- | --- | --- | --- | --- | --- |
| **First Author** | **Year** | **Category** | **Sample Size*** | **Mean Age (y) (mean±SD)** | **Outcomes** | **Significance** | **Funding source** | ***Author COI*** |
| Cain et al^32^ | 2017 | Balance training | 4/7:7/4 | E:(16.45±0.93) C:(16.55±1.29) | Time required to complete 10 lateral jumps | YES | No external funding | None declared |
| Cain et al (1)^33^ | 2020 | Balance training | 8/2:4/7 | E:(16.40±0.97) C:(16.45±1.04) | Time required to complete 10 lateral jumps and 10 8-figure jump | NO | Funding Agency | No conflict of interest |
| Linens et al^34^ | 2016 | Balance training | 17:17 | E:(22.94±2.77) C:(23.18±3.64) | Time required to complete 10 lateral jumps and 10 8-figure jump | YES | Funding Agency | No conflict of interest |
| Park et al (2)^11^ | 2024 | Balance training | 17/0:17/0 | E:(14.1±0.7) C:( 14.4±0.7) | Time required to complete 30-cm by lateral jumps | YES | No external funding | No conflict of interest |
| Cain et al (2)^33^ | 2020 | Strength training | 5/7:4/7 | E:(16.42±1.00) C:(16.45±1.04) | Time required to complete 10 lateral jumps and 10 8-figure jump | NO | Funding Agency | No conflict of interest |
| Hall et al (1)^39^ | 2015 | Strength training | 5/8:7/6 | E:(19.7±2.2) C:(20.5±2.1) | Time to complete two 8-figure jumps tests, with the shortest time taken from the two attempts | NO | No external funding | None declared |
| Park et al (1)^11^ | 2024 | Strength training | 17/0:17/0 | E:(14.1±0.7) C:( 14.4±0.7) | Time required to complete 30-cm by lateral jumps | YES | No external funding | No conflict of interest |
| Jiang et al^44^ | 2022 | 3D training | 26:25 | E:(21.8±1.6) C:(22.5±1.5) | Time required to complete a 6-meter jump test, with the shortest time taken from two attempts | NO | No external funding | No conflict of interest |
| Naderi et al^20^ | 2025 | 3D training | 13/8:14/8 | E:(35.5±9.7) C:(30.9±9.5) | Time to complete 5-m distance，with the shortest time taken from the two attempts | YES | No external funding | None declared |
| Hall et al (2)^39^ | 2015 | Neuromuscular control training | 5/8:7/6 | E:(18.9±1.3) C:(20.5±2.1) | Time to complete two 8-figure jumps tests, with the shortest time taken from the two attempts | NO | No external funding | None declared |

| **L.** | | | | | | |  |  |
| --- | --- | --- | --- | --- | --- | --- | --- | --- |
| **First Author** | **Year** | **Category** | **Sample Size*** | **Mean Age (y) (mean±SD)** | **Outcomes** | **Significance** | **Funding source** | ***Author COI*** |
| Jiang et al^44^ | 2022 | 3D training | 26:25 | E:(21.8±1.6) C:(22.5±1.5) | Total jump distance | YES | No external funding | No conflict of interest |
| Melam et al^41^ | 2018 | Strength training | 15:15 | E:(21.0±2.2) C:(21.3±2.3) | Jump distance | NO | No external funding | None declared |
| Shamseddini et al^29^ | 2021 | Vibration training | 8:5 | E:(35.83±12.08) C:(38.30±10.49) | Maximum jump distance | NO | Funding Agency | No conflict of interest |
| Gao et al^58^ | 2020 | Vibration training | 0/14:0/12 | E:(20±0.7) C:(19±1.1) | Maximum jump distance | NO | Funding Agency | None declared |

VAS: visual analogue scale test; E: Experimental group; C: Control group; CAIT: Cumberland Ankle Instability Tool; AJFAT: Ankle Joint Functional Assessment Tool; SEBT: Star Excursion Balance Test; mSEBT: modified Star Excursion Balance Test; * The presentation of the sample size is as follows: Male in the experimental group / Female in the experimental group: Male in the control group / Female in the control group. Among these 19 articles^1,4,10,12,17,19,23,24,26,28,29,34,36,41,44,45,48,52,54^, the gender information was not reported. The presented data was the total number of participants in the experimental group versus the total number of participants in the control group.

# **Table S2. Egger's test results of included article**

| **Outcomes** | **t** | **p** | **95%CI** |
| --- | --- | --- | --- |
| Joint position sense | 1.40 | 0.20 | -2.78 to 11.74 |
| Dynamic balance | 1.18 | 0.24 | -0.66 to 2.57 |
| Patient-reported functional impairment and stability | 1.79 | 0.08 | -0.03 to 0.48 |
| Concentric inversion muscular strength | 1.33 | 0.20 | -0.19 to 0.85 |
| Concentric eversion muscular strength | 0.72 | 0.48 | -0.09 to 0.18 |

# **Table S3. Quality rating of included article**

| Study | 1 | 2 | 3 | 4 | 5 | 6 | 7 | 8 | 9 | 10 | 11 | Total | Overall Quality |
| --- | --- | --- | --- | --- | --- | --- | --- | --- | --- | --- | --- | --- | --- |
| Balance training | | | | | | | | | | | | | |
| Bernier et al, 1998 | Y | Y | Y | N | N | N | N | Y | Y | Y | Y | 6 | Good |
| McKeon et al, 2008 | Y | Y | Y | Y | N | N | N | N | Y | Y | Y | 6 | Good |
| Jain et al, ,2014 | Y | Y | Y | Y | Y | N | Y | Y | Y | Y | Y | 9 | Excellent |
| Linens et al, 2016 | Y | Y | N | Y | N | N | N | N | Y | Y | Y | 5 | Fair |
| Cain et al, 2017 | Y | Y | N | Y | N | N | N | Y | Y | Y | Y | 6 | Good |
| Sierra-Guzm et al, 2018 | Y | Y | Y | Y | Y | Y | Y | Y | Y | Y | Y | 10 | Excellent |
| Deussen et al, 2018 | Y | Y | Y | Y | Y | Y | Y | Y | Y | Y | Y | 10 | Excellent |
| Cain et al, 2020 | Y | Y | Y | Y | Y | Y | N | Y | Y | Y | Y | 9 | Excellent |
| Sun et al, 2020 | Y | Y | Y | Y | N | N | N | Y | Y | Y | Y | 7 | Good |
| Kim et al, 2021 | Y | Y | N | N | N | N | Y | Y | Y | Y | Y | 6 | Good |
| Taghavi Asl et al, 2022 | Y | Y | Y | Y | N | N | Y | Y | Y | Y | Y | 8 | Good |
| Parlakidis et al, 2024 | Y | Y | N | Y | N | N | N | Y | Y | Y | Y | 6 | Good |
| Sun et al, 2023 | Y | Y | N | Y | Y | N | N | Y | Y | Y | Y | 7 | Good |
| Wang et al, 2023 | Y | Y | N | Y | Y | N | N | Y | Y | Y | Y | 7 | Good |
| Cui et al, 2022 | Y | Y | N | Y | N | N | N | Y | Y | Y | Y | 6 | Good |
| Fan et al, 2022 | Y | Y | N | Y | N | N | N | Y | Y | Y | Y | 6 | Good |
| Zhao et al, 2022 | Y | Y | N | Y | N | N | N | N | Y | Y | Y | 5 | Fair |
| Fu et al, 2020 | Y | Y | N | Y | N | N | N | Y | Y | Y | Y | 6 | Good |
| Hua et al, 2017 | Y | Y | N | Y | N | N | N | Y | Y | Y | Y | 6 | Good |
| Park et al, 2024 | Y | Y | Y | Y | Y | Y | Y | Y | Y | Y | Y | 10 | Excellent |
| Reyes et al, 2024 | Y | Y | N | Y | N | N | N | Y | Y | Y | Y | 6 | Good |
| Average scores |  | | | | | | | | | | | 7 | Good |
| Vibration training | | | | | | | | | | | | | |
| Cloak et al, 2010 | Y | Y | N | Y | N | N | N | N | Y | Y | Y | 5 | Fair |
| Sierra-Guzm et al, 2018 | Y | Y | Y | Y | Y | Y | Y | Y | Y | Y | Y | 10 | Excellent |
| Kim et al, 2019 | Y | Y | Y | Y | Y | N | N | N | Y | Y | Y | 7 | Good |
| Sun et al, 2020 | Y | Y | Y | Y | N | N | N | Y | Y | Y | Y | 7 | Good |
| Shamseddini et al, 2021 | Y | Y | Y | Y | Y | N | Y | Y | Y | Y | Y | 9 | Excellent |
| Astorino et al, 2021 | Y | Y | N | Y | Y | N | N | Y | Y | Y | Y | 7 | Good |
| Gao et al, 2020 | Y | Y | N | Y | N | N | N | N | Y | Y | Y | 5 | Fair |
| Tang et al, 2017 | Y | Y | N | Y | N | N | N | Y | Y | Y | Y | 5 | Fair |
| Liu et al, 2019 | Y | Y | N | N | N | N | N | Y | Y | Y | Y | 5 | Fair |
| Average scores |  | | | | | | | | | | | 6.67 | Good |
| Strength training | | | | | | | | | | | | | |
| Smith et al, 2012 | Y | Y | N | Y | N | N | N | Y | Y | Y | Y | 6 | Good |
| Huang et al, ,2014 | Y | Y | N | Y | N | N | Y | Y | Y | Y | Y | 7 | Good |
| Hall et al, 2015 | Y | Y | N | Y | N | N | N | Y | Y | Y | Y | 6 | Good |
| Melam et al, 2018 | Y | Y | N | Y | N | N | N | Y | Y | Y | Y | 6 | Good |
| Smith et al, 2018 | Y | Y | N | Y | N | N | N | Y | Y | Y | Y | 6 | Good |
| Cain et al, 2020 | Y | Y | Y | Y | Y | Y | N | Y | Y | Y | Y | 9 | Excellent |
| Cruz-Diaz et al, 2020 | Y | Y | N | Y | Y | N | Y | Y | Y | Y | Y | 8 | Good |
| Kim et al, 2022 | Y | Y | N | Y | N | N | N | Y | Y | Y | Y | 6 | Good |
| Park et al, 2023 | Y | Y | N | Y | N | N | N | N | Y | Y | Y | 5 | Fair |
| Zhang et al, 2021 | Y | Y | N | Y | N | N | N | Y | Y | Y | Y | 6 | Good |
| Zhou et al, 2021 | Y | Y | N | Y | N | N | N | Y | Y | Y | Y | 6 | Good |
| Zhou et al, 2018 | Y | Y | Y | Y | Y | Y | Y | Y | Y | Y | Y | 10 | Excellent |
| Luo et al, 2017 | Y | Y | N | N | N | N | N | Y | Y | Y | Y | 5 | Fair |
| Park et al, 2024 | Y | Y | Y | Y | Y | Y | Y | Y | Y | Y | Y | 10 | Excellent |
| Average scores |  | | | | | | | | | | | 6.86 | Good |
| 3D training | | | | | | | | | | | | | |
| Cruz-Díaz et al, 2020 | Y | Y | N | Y | Y | N | Y | Y | Y | Y | Y | 8 | Good |
| Jiang et al, 2022 | Y | Y | N | Y | N | N | N | Y | Y | Y | Y | 6 | Good |
| Li et al, 2023 | Y | Y | Y | Y | N | N | Y | Y | Y | Y | Y | 8 | Good |
| Yang et al, 2022 | Y | Y | N | Y | N | N | N | N | Y | Y | Y | 5 | Fair |
| Wang et al, 2019 | Y | Y | Y | Y | Y | Y | Y | N | Y | Y | Y | 9 | Excellent |
| Nader et al, 2025 | Y | Y | Y | Y | Y | N | Y | Y | Y | Y | Y | 9 | Excellent |
| Xu et al, 2024 | Y | Y | N | Y | N | N | N | Y | Y | Y | Y | 6 | Good |
| Li et al, 2024 | Y | Y | N | Y | N | N | Y | Y | Y | Y | Y | 7 | Good |
| Liu et al, 2024 | Y | Y | N | Y | N | N | N | Y | Y | Y | Y | 6 | Good |
| Average scores |  |  |  |  |  |  |  |  |  |  |  | 7.11 | Good |
| Neruomusclar control training | | | | | | | | | | | | | |
| Hall et al, 2015 | Y | Y | N | Y | N | N | N | Y | Y | Y | Y | 6 | Good |
| Shih et al, 2018 | Y | Y | N | Y | N | N | N | Y | Y | Y | Y | 6 | Good |
| Lbrahim et al, 2020 | Y | Y | Y | Y | Y | N | N | Y | Y | Y | Y | 8 | Good |
| Kim et al, 2022 | Y | Y | N | Y | N | N | N | Y | Y | Y | Y | 6 | Good |
| Liu et al, 2022 | Y | Y | N | N | N | N | N | Y | Y | Y | Y | 5 | Fair |
| Yin et al, 2023 | Y | Y | N | Y | N | N | N | Y | Y | Y | Y | 6 | Good |
| Yuan et al, 2023 | Y | Y | N | Y | N | N | N | Y | Y | Y | Y | 6 | Good |
| Average scores |  | | | | | | | | | | | 6.14 | Good |
| Stroboscopic vision training | | | | | | | | | | | | | |
| Kim et al, 2021 | Y | Y | N | N | N | N | Y | Y | Y | Y | Y | 6 | Good |
| Wu et al, 2024 | Y | Y | N | Y | N | N | Y | Y | Y | Y | Y | 7 | Good |
| Sun et al, 2023 | Y | Y | N | Y | Y | N | N | Y | Y | Y | Y | 7 | Good |
| Average scores |  | | | | | | | | | | | 6.67 | Good |
| Proprioceptive training | | | | | | | | | | | | | |
| Astorino et al, 2021 | Y | Y | N | Y | Y | N | N | Y | Y | Y | Y | 7 | Good |
| Park et al, 2023 | Y | Y | N | Y | N | N | N | N | Y | Y | Y | 5 | Fair |
| Liang et al, 2022 | Y | Y | N | Y | N | N | N | N | Y | Y | Y | 5 | Fair |
| Liang et al, 2019 | Y | Y | N | Y | N | N | N | N | Y | Y | Y | 5 | Fair |
| Average scores |  | | | | | | | | | | | 5.5 | Fair |
| Other type | | | | | | | | | | | | | |
| Deussen et al, 2018 | Y | Y | Y | Y | Y | Y | Y | Y | Y | Y | Y | 10 | Excellent |
| Bagherian et al, 2019 | Y | Y | N | Y | N | N | N | Y | Y | Y | Y | 6 | Good |
| Average scores |  | | | | | | | | | | | 8 | Good |

Y: yes; N: no. PEDro scale criteria. 1: Eligibility criteria were speciﬁed. 2: Subjects were randomly allocated to groups (in a crossover study, subjects were randomly allocated an order in which treatments were received). 3: Allocation was concealed. 4: The groups were similar at baseline regarding most important prognostic indicators. 5: There was blinding of all subjects. 6: There was blinding of all therapists/researchers who administered the therapy/protocol. 7: There was blinding of all assessors who measured at least one key outcome. 8: Measures of at least one key outcome were obtained from more than 85% of the subjects that were initially allocated to groups. 9: All subjects for whom outcome measures were available received the treatment or control condition as allocated or, where this was not the case, data for at least one key outcome were analyzed using “intention to treat.” 10: The results of between-group statistical comparisons were reported for at least one key outcome. 11: The study provided both point measures and measures of variability for at least one key outcome.

# **Table S4. Outcomes of certainty of evidence**

| **Grade framework** |  |  |  |  |
| --- | --- | --- | --- | --- |
| **Outcomes** | **No of Participants (studies)** Follow up | **Quality of the evidence** (GRADE) | **Comments** | **Anticipated absolute effects** |
|  |  |  |  |  |
|  |  |  |  | **Risk difference with Intervention** (95% CI) |
| **Joint position sense** | 335 (11 studies) | ⊕⊕⊕⊝ **MODERATE**^1^ due to inconsistency |  | The mean joint position sense in the intervention groups was **0.66 lower** (1.21 to 0.11 lower) |
| **Joint position sense - Vibration training** | 75 (3 studies) | ⊕⊕⊕⊕ **HIGH** |  | The mean joint position sense - vibration training in the intervention groups was **0.29 lower** (1.08 lower to 0.49 higher) |
| **Joint position sense - Balance training** | 169 (5 studies) | ⊕⊕⊕⊕ **HIGH** |  | The mean joint position sense - balance training in the intervention groups was **1.27 lower** (2.09 to 0.45 lower) |
| **Joint position sense - Stroboscopic vision training** | 21 (1) | See comment | Only one article | The mean joint position sense - stroboscopic vision training in the intervention groups was **0.06 higher** (0.02 to 0.1 higher) |
| **Joint position sense - 3D training** | 30 (1 study) | See comment | Only one article | The mean joint position sense - 3d training in the intervention groups was **0.05 lower** (0.56 lower to 0.46 higher) |
| **Joint position sense - Other** | 40 (1 study) | See comment | Only one article | The mean joint position sense - other in the intervention groups was **1.6 lower** (2.23 to 0.97 lower) |
| **Concentric inversion muscular strength** | 544 (20 studies) | ⊕⊕⊕⊕ **HIGH** |  | The mean concentric inversion muscular strength in the intervention groups was **0.48 standard deviations higher** (0.31 to 0.66 higher) |
| **Concentric inversion muscular strength - Vibration training** | 95 (4 studies) | ⊕⊕⊕⊝ **MODERATE**^1^ due to inconsistency |  | The mean concentric inversion muscular strength - vibration training in the intervention groups was **0.11 standard deviations higher** (0.31 lower to 0.53 higher) |
| **Concentric inversion muscular strength - Balance training** | 122 (5 studies) | ⊕⊕⊕⊕ **HIGH** |  | The mean concentric inversion muscular strength - balance training in the intervention groups was **0.42 standard deviations higher** (0.05 to 0.79 higher) |
| **Concentric inversion muscular strength - Strength training** | 60 (2 studies) | ⊕⊕⊝⊝ **LOW**^1,2^ due to risk of bias, inconsistency |  | The mean concentric inversion muscular strength - strength training in the intervention groups was **0.85 standard deviations higher** (0.31 to 1.4 higher) |
| **Concentric inversion muscular strength - 3D training** | 115 (3 studies) | ⊕⊕⊕⊝ **MODERATE**^1^ due to inconsistency |  | The mean concentric inversion muscular strength - 3d training in the intervention groups was **0.19 standard deviations higher** (0.18 lower to 0.56 higher) |
| **Concentric inversion muscular strength - Neuromuscular training** | 52 (2 studies) | ⊕⊕⊕⊕ **HIGH** |  | The mean concentric inversion muscular strength - neuromuscular training in the intervention groups was **0.71 standard deviations higher** (0.13 to 1.28 higher) |
| **Concentric inversion muscular strength - Proprioceptive training** | 50 (2 studies) | ⊕⊕⊕⊝ **MODERATE**^2^ due to risk of bias |  | The mean concentric inversion muscular strength - proprioceptive training in the intervention groups was **0.85 standard deviations higher** (0.27 to 1.43 higher) |
| **Concentric inversion muscular strength - Other** | 50 (2 studies) | ⊕⊕⊕⊕ **HIGH** |  | The mean concentric inversion muscular strength - other in the intervention groups was **1.16 standard deviations higher** (0.54 to 1.77 higher) |
| **Eccentric inversion muscular strength** | 120 (4 studies) | ⊕⊕⊕⊕ **HIGH** |  | The mean eccentric inversion muscular strength in the intervention groups was **1.44 higher** (1.27 lower to 4.15 higher) |
| **Eccentric inversion muscular strength - Vibration training** | 45 (2 studies) | ⊕⊕⊕⊕ **HIGH** |  | The mean eccentric inversion muscular strength - vibration training in the intervention groups was **0.64 lower** (8.16 lower to 6.89 higher) |
| **Eccentric inversion muscular strength - Balance training** | 75 (2 studies) | ⊕⊕⊕⊕ **HIGH** |  | The mean eccentric inversion muscular strength - balance training in the intervention groups was **1.75 higher** (1.15 lower to 4.66 higher) |
| **Concentric eversion muscular strength** | 606 (22 studies) | ⊕⊕⊕⊝ **MODERATE**^1^ due to inconsistency |  | The mean concentric eversion muscular strength in the intervention groups was **0.48 standard deviations higher** (0.17 to 0.79 higher) |
| **Concentric eversion muscular strength - Vibration training** | 120 (5 studies) | ⊕⊕⊕⊕ **HIGH** |  | The mean concentric eversion muscular strength - vibration training in the intervention groups was **0.14 standard deviations lower** (0.64 lower to 0.35 higher) |
| **Concentric eversion muscular strength- Balance training** | 147 (6 studies) | ⊕⊕⊕⊕ **HIGH** |  | The mean concentric eversion muscular strength- balance training in the intervention groups was **0.6 standard deviations higher** (0.26 to 0.94 higher) |
| **Concentric eversion muscular strength - Strength training** | 66 (2 studies) | ⊕⊕⊝⊝ **LOW**^1,2^ due to risk of bias, inconsistency |  | The mean concentric eversion muscular strength - strength training in the intervention groups was **0.81 standard deviations higher** (0.01 lower to 1.62 higher) |
| **Concentric eversion muscular strength - 3D training** | 115 (3 studies) | ⊕⊕⊝⊝ **LOW**^3^ due to inconsistency |  | The mean concentric eversion muscular strength - 3d training in the intervention groups was **0.14 standard deviations higher** (0.71 lower to 0.99 higher) |
| **Concentric eversion muscular strength - Neuromuscular training** | 58 (2 studies) | ⊕⊕⊕⊝ **MODERATE**^1^ due to inconsistency |  | The mean concentric eversion muscular strength - neuromuscular training in the intervention groups was **0.66 standard deviations higher** (0.21 lower to 1.52 higher) |
| **Concentric eversion muscular strength - Proprioceptive training** | 50 (2 studies) | ⊕⊝⊝⊝ **VERY LOW**^2,3^ due to risk of bias, inconsistency |  | The mean concentric eversion muscular strength - proprioceptive training in the intervention groups was **1.05 standard deviations higher** (0.57 lower to 2.68 higher) |
| **Concentric eversion muscular strength - Other** | 50 (2 studies) | ⊕⊕⊕⊝ **MODERATE**^1^ due to inconsistency |  | The mean concentric eversion muscular strength - other in the intervention groups was **1.38 standard deviations higher** (0.07 to 2.69 higher) |
| **Eccentric eversion muscular strength** | 170 (6 studies) | ⊕⊕⊕⊕ **HIGH** |  | The mean eccentric eversion muscular strength in the intervention groups was **1.17 higher** (1.24 lower to 3.58 higher) |
| **Eccentric eversion muscular strength - Vibration training** | 70 (3 studies) | ⊕⊕⊕⊕ **HIGH** |  | The mean eccentric eversion muscular strength - vibration training in the intervention groups was **0.49 higher** (4.72 lower to 5.71 higher) |
| **Eccentric eversion muscular strength - Balance training** | 100 (3 studies) | ⊕⊕⊕⊕ **HIGH** |  | The mean eccentric eversion muscular strength - balance training in the intervention groups was **1.35 higher** (1.36 lower to 3.58 higher) |
| **Dynamic balance** | 1585 (51 studies) | ⊕⊕⊕⊕ **HIGH** |  | The mean dynamic balance in the intervention groups was **0.59 standard deviations higher** (0.46 to 0.73 higher) |
| **Dynamic balance - Vibration training** | 127 (5 studies) | ⊕⊕⊕⊕ **HIGH** |  | The mean dynamic balance - vibration training in the intervention groups was **0.51 standard deviations higher** (0.14to 0.87 higher) |
| **Dynamic balance - Balance training** | 386 (14 studies) | ⊕⊕⊕⊝ **MODERATE**^1^ due to inconsistency |  | The mean dynamic balance - balance training in the intervention groups was **0.70 standard deviations higher** (0.35 to 1.06 higher) |
| **Dynamic balance - Strength training** | 466 (13 studies) | ⊕⊕⊕⊕ **HIGH** |  | The mean dynamic balance - strength training in the intervention groups was **0.57 standard deviations higher** (0.38 to 0.76 higher) |
| **Dynamic balance - 3D training** | 282 (7 studies) | ⊕⊕⊕⊝ **MODERATE**^1^ due to inconsistency |  | The mean dynamic balance - 3d training in the intervention groups was **0.48 standard deviations higher** (0.07 to 0.88 higher) |
| **Dynamic balance - Neuromuscular training** | 152 (6 studies) | ⊕⊕⊕⊕ **HIGH** |  | The mean dynamic balance - neuromuscular training in the intervention groups was **0.55 standard deviations higher** (0.14 to 0.95 higher) |
| **Dynamic balance - Stroboscopic vision training** | 94 (3 studies) | ⊕⊕⊕⊝ **MODERATE**^1^ due to inconsistency |  | The mean dynamic balance - stroboscopic vision training in the intervention groups was **0.85 standard deviations higher** (0.07 to 1.64 higher) |
| **Dynamic balance - Proprioceptive training** | 76 (3 studies) | ⊕⊝⊝⊝ **VERY LOW**^1,4^ due to risk of bias, inconsistency |  | The mean dynamic balance - proprioceptive training in the intervention groups was **0.46 standard deviations higher** (0.11 lower to 1.03 higher) |
| **Dynamic balance - Other** | 40 (1 study) | See comment | Only one article | The mean dynamic balance - other in the intervention groups was **1.26 standard deviations higher** (0.58 to 1.95 higher) |
| **Functional performance (based on the time completing the test)** | 276 (10 studies) | ⊕⊕⊝⊝ **LOW**^3^ due to inconsistency |  | The mean functional performance (based on the time completing the test) in the intervention groups was **1.27 lower** (2.21 to 0.33 lower) |
| **Functional performance (based on the time completing the test) - Balance training** | 98 (4 studies) | ⊕⊕⊕⊕ **HIGH** |  | The mean functional performance (based on the time completing the test) - balance training in the intervention groups was **2.56 lower** (4.57 to 0.54 lower) |
| **Functional performance (based on the time completing the test)- Strength training** | 64 (3 studies) | ⊕⊕⊕⊕ **HIGH** |  | The mean functional performance (based on the time completing the test)- strength training in the intervention groups was **1.38 lower** (2.40 lower to 0.37 higher) |
| **Functional performance (based on the time completing the test)- 3D training** | 94 (2 studies) | ⊕⊝⊝⊝ **VERY LOW**^2,3^ due to risk of bias, inconsistency |  | The mean functional performance (based on the time completing the test)- 3d training in the intervention groups was **0.82 lower** (2.79 lower to 1.14 higher) |
| **Functional performance (based on the time completing the test) - Neuromuscular training** | 20 (1) | See comment | Only one article | The mean functional performance (based on the time completing the test) - neuromuscular training in the intervention groups was **0.1 lower** (2.25 lower to 2.05 higher) |
| **Functional performance (based on the distance completing the test)** | 120 (4 studies) | ⊕⊕⊕⊕ **HIGH** |  | The mean functional performance (based on the distance completing the test) in the intervention groups was **0.38 standard deviations higher** (0.01 to 0.74 higher) |
| **Patient-reported functional impairment and stability** | 999 (31 studies) | ⊕⊕⊕⊝ **MODERATE**^1^ due to inconsistency |  | The mean patient-reported functional impairment and stability in the intervention groups was **0.77 standard deviations higher** (0.5 to 1.03 higher) |
| **Patient-reported functional impairment and stability - Vibration training** | 70 (2 studies) | ⊕⊕⊕⊕ **HIGH** |  | The mean patient-reported functional impairment and stability - vibration training in the intervention groups was **0.04 standard deviations lower** (0.51 lower to 0.43 higher) |
| **Patient-reported functional impairment and stability - Balance training** | 229 (7 studies) | ⊕⊕⊕⊕ **HIGH** |  | The mean patient-reported functional impairment and stability - balance training in the intervention groups was **0.56 standard deviations higher** (0.22 to 0.89 higher) |
| **Patient-reported functional impairment and stability - Strength training** | 261 (7 studies) | ⊕⊕⊕⊝ **MODERATE**^1^ due to inconsistency |  | The mean patient-reported functional impairment and stability - strength training in the intervention groups was **0.71 standard deviations higher** (0.2 to 1.22 higher) |
| **Patient-reported functional impairment and stability - 3D training** | 203 (6 studies) | ⊕⊕⊕⊝ **MODERATE**^1^ due to inconsistency |  | The mean patient-reported functional impairment and stability - 3d training in the intervention groups was **0.63 standard deviations higher** (0.09 to 1.17 higher) |
| **Patient-reported functional impairment and stability - Neuromuscular training** | 142 (5 studies) | ⊕⊕⊝⊝ **LOW**^3^ due to inconsistency |  | The mean patient-reported functional impairment and stability - neuromuscular training in the intervention groups was **1.73 standard deviations higher** (0.67 to 2.79 higher) |
| **Patient-reported functional impairment and stability - Stroboscopic vision training** | 96 (3 studies) | ⊕⊕⊕⊕ **HIGH** |  | The mean patient-reported functional impairment and stability - stroboscopic vision training in the intervention groups was **0.55 standard deviations higher** (0.05 to 1.05 higher) |
| **Patient-reported functional impairment and stability - Proprioceptive training** | 18 (1) | See comment | Only one article | The mean patient-reported functional impairment and stability - proprioceptive training in the intervention groups was **2.52 standard deviations higher** (1.07 to 3.97 higher) |
| **Pain** | 66 (3 studies) | ⊕⊕⊝⊝ **LOW**^4^ due to risk of bias |  | The mean pain in the intervention groups was **0.64 lower** (1.17 to 0.1 lower) |
| **Muscular reaction time** | 117 (3 studies) | ⊕⊕⊕⊕ **HIGH** |  | The mean muscular reaction time in the intervention groups was **8.19 lower** (10.91 to 5.46 lower) |
| **Force sense** | 84 (3 studies) | ⊕⊕⊝⊝ **LOW**^4^ due to risk of bias |  | The mean force sense in the intervention groups was **0.4 standard deviations lower** (0.84 lower to 0.03 higher) |
| *The basis for the **assumed risk** (e.g. the median control group risk across studies) is provided in footnotes. The **corresponding risk** (and its 95% confidence interval) is based on the assumed risk in the comparison group and the **relative effect** of the intervention (and its 95% CI).  **CI:** Confidence interval; | | | | |
| GRADE Working Group grades of evidence **High quality:** Further research is very unlikely to change our confidence in the estimate of effect.  **Moderate quality:** Further research is likely to have an important impact on our confidence in the estimate of effect and may change the estimate. | | | | |
| **Low quality:** Further research is very likely to have an important impact on our confidence in the estimate of effect and is likely to change the estimate. **Very low quality:** We are very uncertain about the estimate. | | | | |
| **^1^ 50%＜I2＜75% ^2^ One article was high risk of bias ^3^ I²＞75% ^4^ Two articles were high risk of bias** | | | | |

# **Figure S1. Publication bias of included articles: A- Joint position sense; B- Dynamic balance; C- Patient-reported functional impairment and stability; D- Concentric inversion muscular strength; E- Concentric eversion muscular strength**

**B**

**A**

**E**

**D**

**C**

# **Figure S2. Sensitive analysis of included articles: A-Pain; B-Joint position sense; C-Force sense; D-Muscular reaction time; E-Patient-reported functional impairment and stability; F-Dynamic balance; G-Concentric inversion muscular strength; H**-**Concentric eversion muscular strength; I-Eccentric inversion muscular strength; J-Eccentric eversion muscular strength; K-Functional performance (based on the time completing the test); L-Functional performance (based on the distance completing the test)**


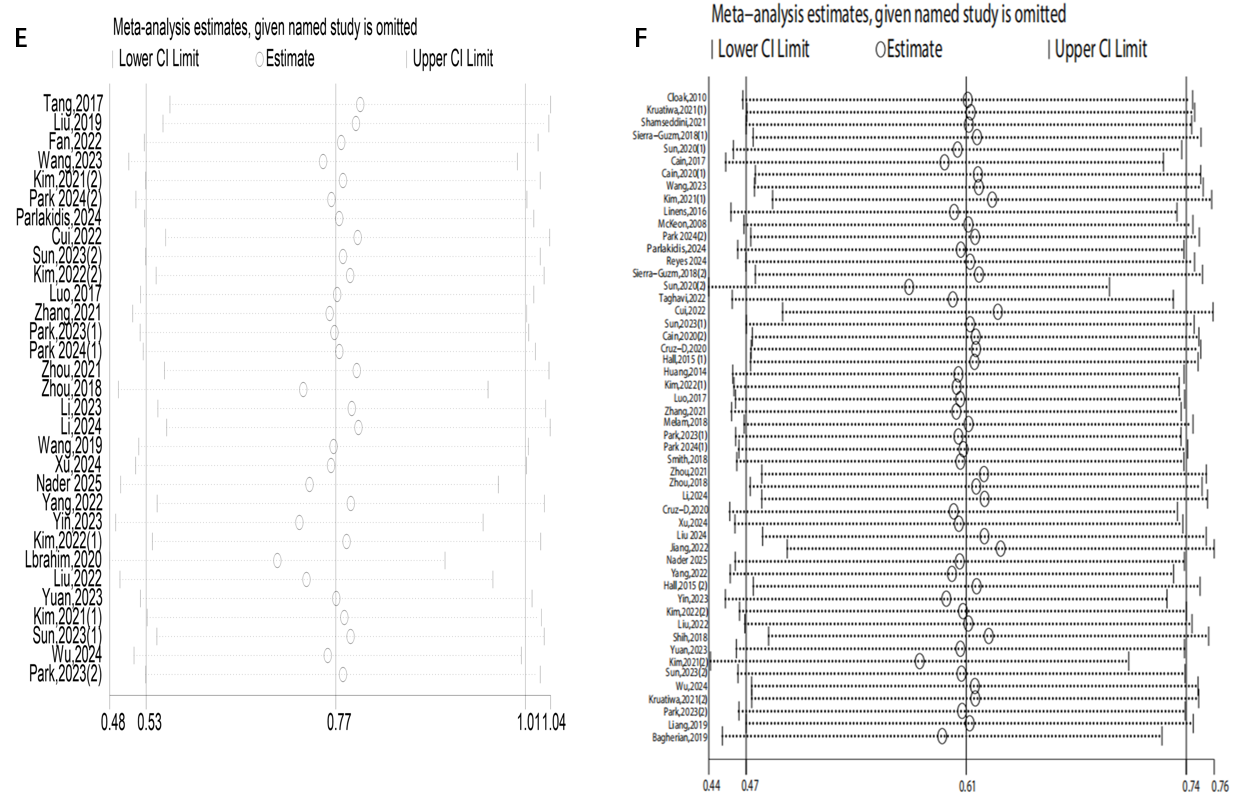

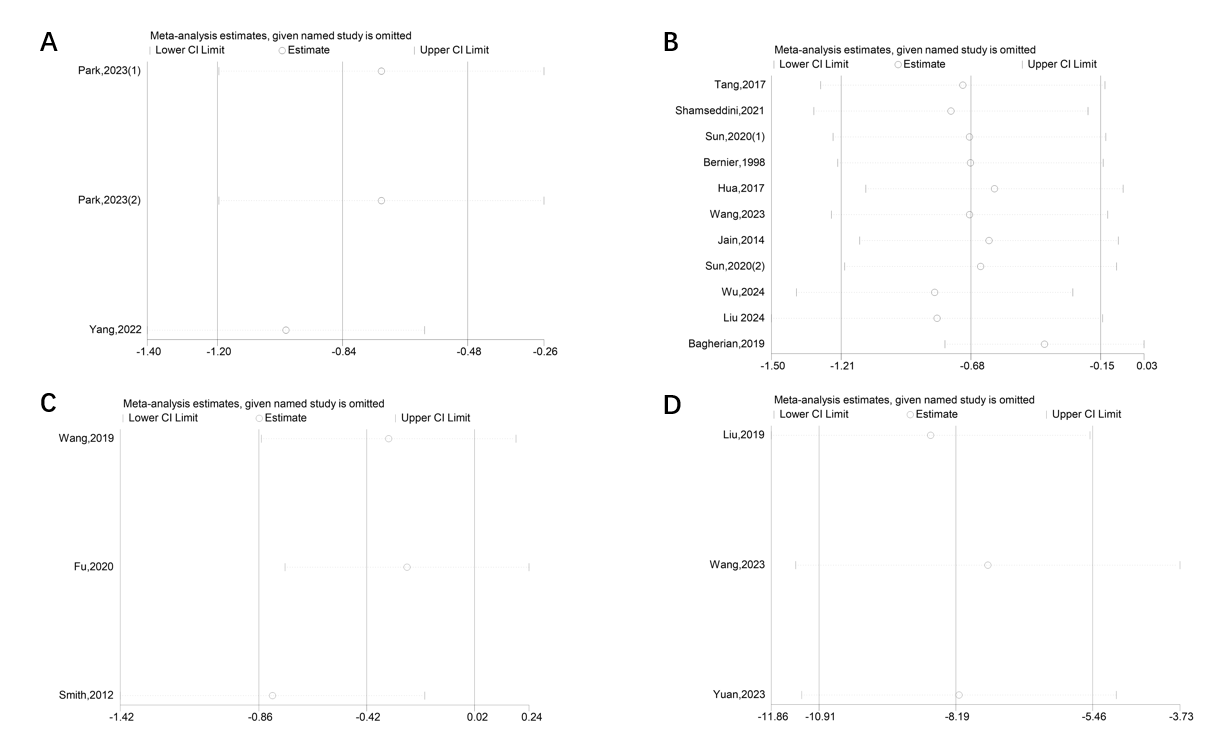


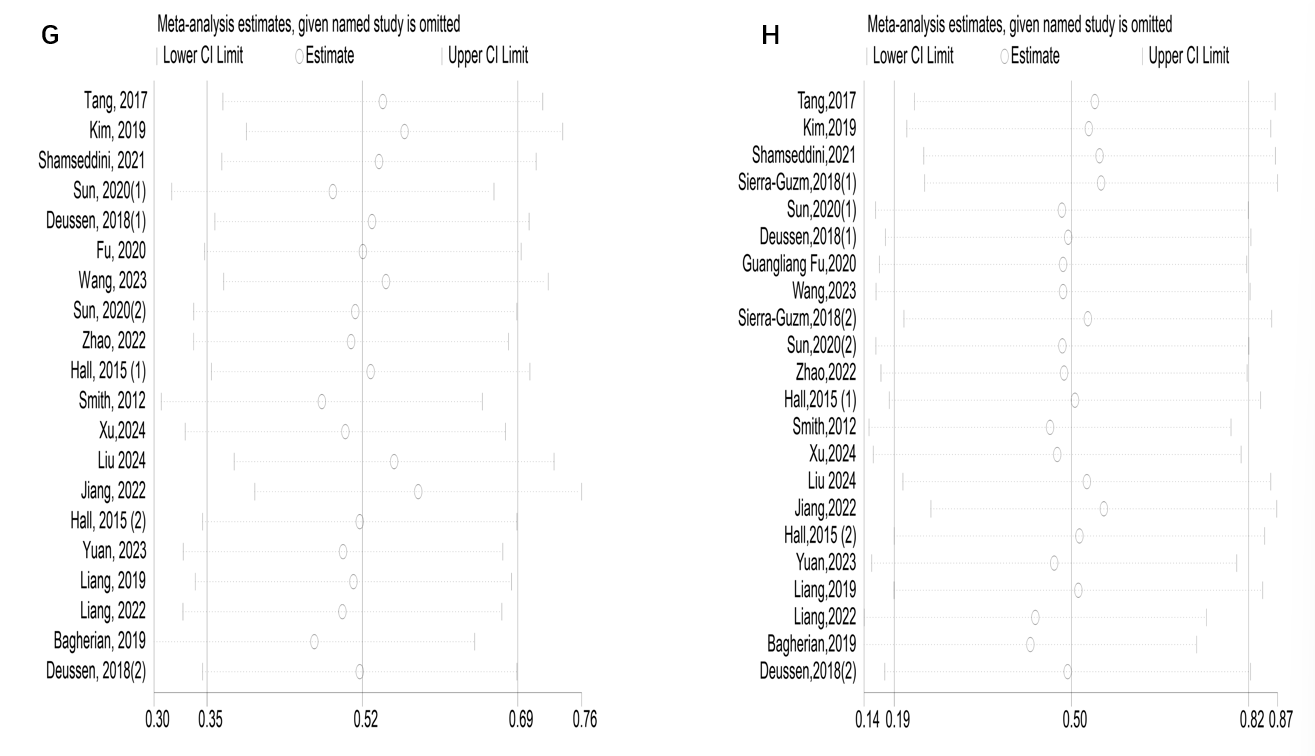

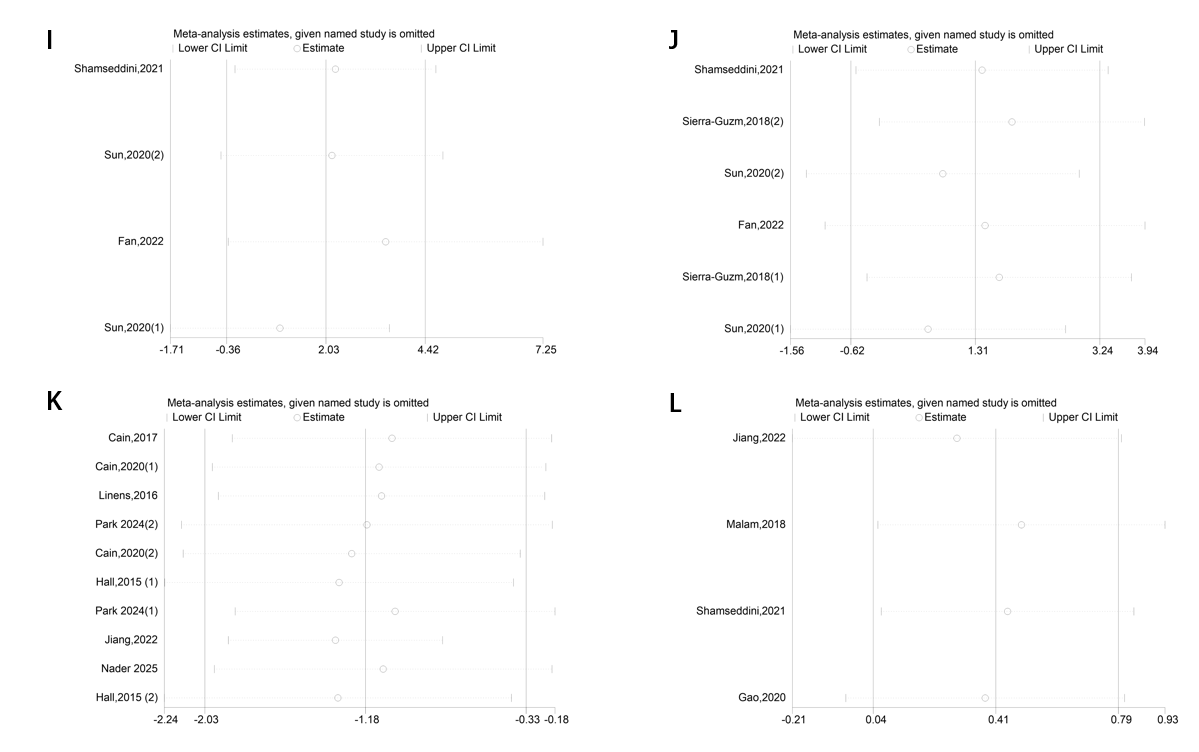


# **Reference**

1. Park Y J, Cho Y H, Seo T B. Effect of two different exercises on balance, pain and ankle motor function in male college students with chronic ankle instability[J]. 2023.

2. Yang Yan. Research on the Intervention Effect of Functional Training Combined with Rehabilitation Therapy on College Students with CAI [D]. Xi'an Physical Education University, 2022.

3. Tang Chenxi. The Impact of Progressive Frequency Whole-Body Vibration Training with the Same Amplitude on Functional Ankle Instability [D]. Chengdu Sport University, 2017.

4. Liu Chunlong, Zhang Xiaoya, Gao Qi. Surface EMG Study on the Intervention of Vibration Training for Functional Ankle Instability [J]. Sports & Science Technology, 2019, (21): 210-211.

5. Fan hongyu. Comparative Study on Muscle Strength and Balance of NJF and Stability Training in Young Patients with Functional Ankle Instability [D].North China University of Science and Technology, 2022.

6. Wang Hui, Chen Huifang, Jia Yudong, et al. The Impact of Ankle Joint Balance Device Training on Ankle Joint Function and Postural Control Ability in Patients with Functional Ankle Instability [J]. Chinese Rehabilitation, 2023, 38(02): 81-85.

7. Kim, K.-M., Estudillo-Martínez, M. D., Castellote-Caballero, Y., Estepa-Gallego, A. & Cruz-Díaz, D. Short-Term Effects of Balance Training with Stroboscopic Vision for Patients with Chronic Ankle Instability: A Single-Blinded Randomized Controlled Trial. *Int J Environ Res Public Health* **18**, 5364 (2021).

8. Parlakidis, K. *et al.* The Effectiveness of External Verbal Feedback on Balance in Athletes with Chronic Ankle Instability. *JFMK* **9**, 56 (2024).

9. Cui Xianghong, Hu Dawei, Tang Di, et al. Observational Study on the Therapeutic Effect of Adaptive Balance Rehabilitation Training Device on Functional Ankle Instability [J]. Chinese Journal of Convalescent Medicine, 2022, 31(05): 509-512.

10. Sun Xianghong. The Efficacy of Balance Training with Visual Interference on the Rehabilitation of Patients with Chronic Ankle Instability [D]. Wuhan Sports University, 2023.

11. Park, H. S., Oh, J. K., Kim, J. Y. & Yoon, J. H. The Effect of Strength and Balance Training on Kinesiophobia, Ankle Instability, Function, and Performance in Elite Adolescent Soccer Players with Functional Ankle Instability: A Prospective Cluster Randomized Controlled Trial. *jsportscimed* 593–602 (2024)

12. Kim, K.-M., Estepa-Gallego, A., Estudillo-Martínez, M. D., Castellote-Caballero, Y. & Cruz-Díaz, D. Comparative Effects of Neuromuscular- and Strength-Training Protocols on Pathomechanical, Sensory-Perceptual, and Motor-Behavioral Impairments in Patients with Chronic Ankle Instability: Randomized Controlled Trial. *Healthcare* **10**, 1364 (2022).

13. Luo Li, Sun Wudong, Zhao Xianghu, et al. The Effect of Strengthening Training of the Hip Muscle Group on Functional Ankle Instability [J]. Chinese Journal of Rehabilitation Theory and Practice, 2017, 23(10): 1195-1199.

14. Zhang Meiying, Zhao Lei, Li Hui, et al. Therapeutic Effect of Peri-Hip Muscle Strength Training on Functional Ankle Instability and Surface EMG Evaluation [J]. Chinese Journal of Rehabilitation Theory and Practice, 2021, 27(08): 936-942.

15. Zhou Tan. Research on the Intervention Effect of Peri-Hip Muscle Strength Training on Chronic Ankle Instability [D]. Beijing Sport University, 2021.

16. Zhou Yang. The Impact of Hip and Knee Muscle Strengthening on the Rehabilitation of Functional Ankle Instability [D]. Zhejiang Chinese Medical University, 2018.

17. Li Baofang, Li Xiang. The Impact of Chen Style Tai Chi on Proprioception and Balance Function in Patients with Chronic Ankle Instability [C] // Chinese Rehabilitation Medical Association. Proceedings of the 2023 Annual Conference of the Chinese Rehabilitation Medical Association and International Rehabilitation Medical Industry Expo. Fujian University of Traditional Chinese Medicine, School of Rehabilitation Medicine; 2023: 10..

18. Li Baofang, Meng Yingpei, Liu Jun, Lai Jiangong, & Li Xiang. (2024). The impact of Chen-style Tai Chi on balance ability in individuals with chronic ankle instability. Fujian Journal of Traditional Chinese Medicine, 55(08), 37-41.

19. Wang Gang, Zhu Xiaotian. Research on the Intervention Effect of Tai Chi Combined with Rehabilitation Training on Functional Ankle Instability [D]. Xi'an Physical Education University, 2019.

20. Naderi, A. & Ebrahimi, S. Z. Effects of Tai Chi training on functionality, dynamic balance, kinesiophobia, and quality of life in athletes with functional ankle instability. *Research in Sports Medicine* **33**, 48–61 (2025).

21. Xu Guocai, Tang Huiru, Fan Zhiyong, Wang Jiangna, Zhang Cui, & Nie Xiangkun. (2024). The effects of simplified Tai Chi intervention on rehabilitation outcomes and neuromuscular control in individuals with chronic ankle instability. Chinese Journal of Sports Medicine, 43(09), 681-688.

22. Yin Jiesong, Liao Changyan, Zhang Zhuangzhuang. Analysis of the Effect of Proprioceptive Neuromuscular Facilitation Techniques in Treating Functional Ankle Instability [J]. Journal of Nantong University (Medical Edition), 2023, 43(01): 63-66.

23. Ibrahim, A. R. & Abdallah, A. A. A. Dynamic Limit of Stability and Ankle Joint Function Following Neuromuscular Training of Unstable Ankle Joints. A Randomized Controlled Trial. *Fiz Pol* **20**, 68–75 (2020).

24. Liu, H. Effects of neuromuscular treatment on postural balance in athletes recovering from the ankle injury. *Rev Bras Med Esporte* **28**, 517–520 (2022).

25. Yuan Shuqing. The Impact of Six-Week PNF Training on the Stability of Individuals with Functional Ankle Instability [D]. Shandong Institute of Physical Education and Sport, 2023.

26. Wu Yihan, Liu Zhongqiang, Wei Qiaoye, et al. The Impact of Balance Training under Different Visual Conditions on Proprioception in Patients with Chronic Ankle Instability [J]. Chinese Journal of Tissue Engineering Research, 2025, 29(05): 1050-1057.

27. R, C., Am, N., F, C., S, D. & Ma, W. Vibration training improves balance in unstable ankles. *International journal of sports medicine* **31**, (2010).

28. Astorino, T., Baker, J., Brock, S., Dalleck, L., Goulet, E., Gotshall, R., ... & Zhou, B. (2021). Effects of Two Rehabilitation Programs on Dynamic Balance in Athletes with Functional Ankle Instability. Journal of Exercise Physiologyonline, 24(4).

29. Shamseddini Sofla, F., Hadadi, M., Rezaei, I., Azhdari, N. & Sobhani, S. The effect of the combination of whole body vibration and shoe with an unstable surface in chronic ankle instability treatment: a randomized clinical trial. *BMC Sports Sci Med Rehabil* **13**, 28 (2021).

30. Sierra-Guzmán, R., Jiménez-Diaz, F., Ramírez, C., Esteban, P. & Abián-Vicén, J. Whole-Body–Vibration Training and Balance in Recreational Athletes With Chronic Ankle Instability. *Journal of Athletic Training* **53**, 355–363 (2018).

31. Chang, W.-D., Chen, S. & Tsou, Y.-A. Effects of Whole-Body Vibration and Balance Training on Female Athletes with Chronic Ankle Instability. *J Clin Med* **10**, 2380 (2021).

32. Cain, M. S., Garceau, S. W. & Linens, S. W. Effects of a 4-Week Biomechanical Ankle Platform System Protocol on Balance in High School Athletes With Chronic Ankle Instability. *J Sport Rehabil* **26**, 1–7 (2017).

33. Cain, M. S. *et al.* Four-Week Ankle-Rehabilitation Programs in Adolescent Athletes With Chronic Ankle Instability. *Journal of Athletic Training* **55**, 801–810 (2020).

34. Linens, S. W., Ross, S. E. & Arnold, B. L. Wobble Board Rehabilitation for Improving Balance in Ankles With Chronic Instability. *Clinical Journal of Sport Medicine* **26**, 76–82 (2016).

35. McKeon, P. O. *et al.* Balance training improves function and postural control in those with chronic ankle instability. *Med Sci Sports Exerc* **40**, 1810–1819 (2008).

36. Taghavi Asl, A., Shojaedin, S. S. & Hadadnezhad, M. Comparison of effect of wobble board training with and without cognitive intervention on balance, ankle proprioception and jump landing kinetic parameters of men with chronic ankle instability: a randomized control trial. *BMC Musculoskelet Disord* **23**, (2022).

37. Reyes, M. C. *et al.* Cross-education effects of balance training in individuals with chronic ankle instability. *Journal of Bodywork and Movement Therapies* **40**, 1263–1268 (2024).

38. Cruz-Díaz, D., Hita-Contreras, F., Martínez-Amat, A., Aibar-Almazán, A. & Kim, K.-M. Ankle-Joint Self-Mobilization and CrossFit Training in Patients With Chronic Ankle Instability: A Randomized Controlled Trial. *Journal of Athletic Training* **55**, 159–168 (2020).

39. Hall, E. A., Docherty, C. L., Simon, J., Kingma, J. J. & Klossner, J. C. Strength-Training Protocols to Improve Deficits in Participants With Chronic Ankle Instability: A Randomized Controlled Trial. *Journal of Athletic Training* **50**, 36–44 (2015).

40. Cruz-Diaz, D., Lomas-Vega, R., Osuna-Pérez, M. C., Contreras, F. H. & Martínez-Amat, A. Effects of 6 Weeks of Balance Training on Chronic Ankle Instability in Athletes: A Randomized Controlled Trial. *Int J Sports Med* **36**, 754–760 (2015).

41. Melam, G. R. *et al.* Effect of weight-bearing overload using elastic tubing on balance and functional performance in athletes with chronic ankle instability. *Science & Sports* **33**, e229–e236 (2018).

42. Smith, B. I., Curtis, D. & Docherty, C. L. Effects of Hip Strengthening on Neuromuscular Control, Hip Strength, and Self-Reported Functional Deficits in Individuals With Chronic Ankle Instability. *Journal of Sport Rehabilitation* **27**, 364–370 (2018).

43. Cruz-Díaz, D. *et al.* Effects of 12 Weeks of Tai Chi Intervention in Patients With Chronic Ankle Instability: A Randomized Controlled Trial. *Journal of Sport Rehabilitation* **29**, 326–331 (2020).

44. Jiang, Q., Kim, Y. & Choi, M. Kinetic Effects of 6 Weeks’ Pilates or Balance Training in College Soccer Players with Chronic Ankle Instability. *IJERPH* **19**, 12903 (2022).

45. Liu Hao. (2024). The Effect of Tai Chi with Added Focus Intervention on Functional Ankle Instability (Master's thesis, Hebei Normal University).

46. Shih, Y.-F. *et al.* The effect of additional joint mobilization on neuromuscular performance in individuals with functional ankle instability. *Physical Therapy in Sport* **30**, 22–28 (2018).

47. Liang Shanshan. Research on the Impact of Different Intervention Methods on Postural Stability in Individuals with Functional Ankle Instability [D]. Tianjin Sport University, 2019.

48. Bagherian, S., Rahnama, N. & Wikstrom, E. A. Corrective Exercises Improve Movement Efficiency and Sensorimotor Function but Not Fatigue Sensitivity in Chronic Ankle Instability Patients: A Randomized Controlled Trial. *Clinical Journal of Sport Medicine* **29**, 193–202 (2019).

49. Jn, B. & Dh, P. Effect of coordination training on proprioception of the functionally unstable ankle. *The Journal of orthopaedic and sports physical therapy* **27**, (1998).

50. Hua Bing. The Impact of Slip Perturbation Training on Proprioception and Balance Ability in Individuals with Functional Ankle Instability [J]. Journal of Shandong Institute of Physical Education and Sport, 2017, 33(03): 86-91.

51. Jain, T. K. (2014). Objective evaluation of functional ankle instability and balance exercise treatment. Lawrence: University of Kansas.

52. Fu Guangliang. The Impact of Balance Training on Ankle Proprioception and Gait in Individuals with Functional Ankle Instability [D]. Tianjin Sport University, 2020.

53. Bi, S., Cl, D., J, S., J, K. & J, S. Ankle strength and force sense after a progressive, 6-week strength-training program in people with functional ankle instability. *Journal of athletic training* **47**, (2012).

54. Osan University, Kim, S., Kim, Y. & Kim, Y. The Effects of Vibration Exercise after Modified Bröstrom Operation in Soccer Players with Ankle Instability. *JIAPTR* **10**, 1791–1796 (2019).

55. Deussen, S. & Alfuth, M. The influence of sensorimotor training modalities on balance, strength, joint function, and plantar foot sensitivity in recreational athletes with a history of ankle sprain: a randomized controlled pilot study. *Intl J Sports Phys Ther* **13**, 993–1007 (2018).

56. Zhao Tiancheng. Research on the Rehabilitation Effect of Stability Training for High School Male Basketball Players with Ankle Sprains [D]. Tianjin University of Sport, 2022.

57. Liang Shanshan, Meng Qinghua. The effect of proprioceptive training on postural stability and muscle strength recovery after ankle joint injury [J]. Journal of Hebei Institute of Physical Education, 2022, 36(02): 83-88.

58. Gao Xiaojuan, Chu Zhaowei, Li Wenyuan, et al. The Impact of Frequency-Variable Vibration Training on Functional Ankle Instability [J]. Medical Biomechanics, 2020, 35(06): 685-691.
